# Needle Arthroscopy for Bacterial Arthritis of a Native Joint: Surgical Technique for the Shoulder, Elbow, Wrist, Knee, and Ankle Under Local Anesthesia

**DOI:** 10.1016/j.eats.2022.05.011

**Published:** 2022-09-21

**Authors:** Alex B. Walinga, Tobias Stornebrink, Stein J. Janssen, Miki Dalmau-Pastor, Arthur J. Kievit, Gino M.MJ. Kerkhoffs

**Affiliations:** a Amsterdam UMC Location University of Amsterdam, Department of Orthopedic Surgery and Sports Medicine, Amsterdam, The Netherlands; b Amsterdam Movement Sciences, Sports, Musculoskeletal Health, Amsterdam, The Netherlands; c Academic Center for Evidence-based Sports medicine (ACES), Amsterdam, The Netherlands; d Amsterdam Collaboration on Health & Safety in Sports (ACHSS), IOC Research Center, Amsterdam, The Netherlands; e Human Anatomy and Embryology Unit, Department of Pathology and Experimental Therapeutics, School of Medicine and Health Sciences, The University of Barcelona, Barcelona, Spain; f MIFAS by GRECMIP (Minimally Invasive Foot and Ankle Society), Merignac, France

## Abstract

Suspected bacterial arthritis of a native joint requires urgent management to control potential life-threatening sepsis and limit cartilage damage. Diagnosing bacterial arthritis is often challenging and relies on diagnostic tests with low accuracy. A high threshold for surgery poses a risk of undertreatment, whereas a low threshold for surgery could lead to overtreatment with unnecessary invasive and costly procedures. Surgical lavage through arthroscopy or arthrotomy is generally considered standard treatment. Nowadays, needle arthroscopy provides an alternative and potentially less-invasive approach that can safely lower the surgical threshold. Needle arthroscopy can be performed directly upon presentation at the patient's bedside, as it is well tolerated under local anesthesia. Therefore, this Technical Note presents a stepwise guideline for performing standardized needle arthroscopic lavage in patients with (suspected) bacterial arthritis of the shoulder, elbow, wrist, knee, and ankle.

Surgical lavage, arthroscopic or open, followed by antibiotic treatment is considered standard treatment for a bacterial infection of a native joint.^1^ However, the diagnosis of bacterial arthritis is difficult and relies on diagnostic tests with poor-to-moderate accuracy (i.e., specificity and sensitivity).^2^ This results in a practice in which a high threshold for surgery poses a risk of undertreatment for true positives and a low threshold for surgery will result in an unnecessarily invasive and costly procedure for false positives.

Needle arthroscopy under local anesthesia has been proposed as a treatment modality for native joint bacterial arthritis that can be performed directly upon presentation at the patient's bedside, in the emergency department, or in the outpatient clinic.^3^ In a minimally invasive manner, it may address true-positive patients in a timely fashion yet decrease the burden of overtreatment for false-positive cases.

Compared with conventional arthroscopy, needle arthroscopy has various differences. Less equipment is needed, and only small (2-mm) portals are required, which are acceptable for the patient under local anesthesia.^3^ The scope's direction of view is 0°, with a 120° field of view, which may be unfamiliar to many surgeons who are comfortable with the 30° viewing angle in standard arthroscopy. These differences in instrumentation and technology necessitate a modified technique to accommodate thorough, uniform, and safe therapeutic lavage for bacterial arthritis at the patient's bedside. In addition, this technique combines adequate diagnostic joint aspiration followed by direct minimally invasive lavage.

Therefore, the purpose of this article is to provide a surgical guide for performing a standardized needle arthroscopic approach for the treatment of patients with bacterial arthritis of a native shoulder, elbow, wrist, knee, and ankle.

## Surgical Technique (With Video Illustration)

Video 1 demonstrates the surgical technique for each joint. In this section, we first explain the general use of needle arthroscopy as it applies to all joints. Subsequently, joint-specific patient setup, anatomical landmarks, and the inflow and outflow portals are clarified per joint (i.e., shoulder, elbow, wrist, knee, and ankle). Appendix 1 (available at www.arthroscopyjournal.org) includes pocket cards that can be used in clinical practice and depict a standard approach to each joint separately, along with an overview of the general setup and required materials.

### Equipment and Setup

**Fig 1:**
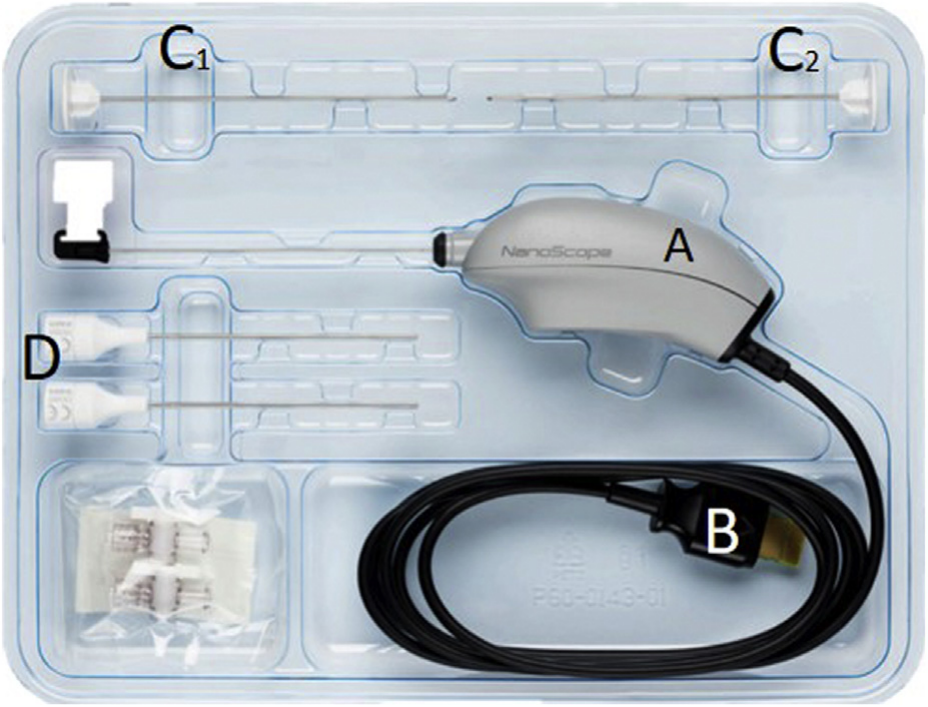
The sterile disposable handpiece set. Number A denotes the handpiece, B the connection cable to the console, C_1_ and C_2_ the sharp and blunt obturators, and D the corresponding sheaths.

**Fig 2:**
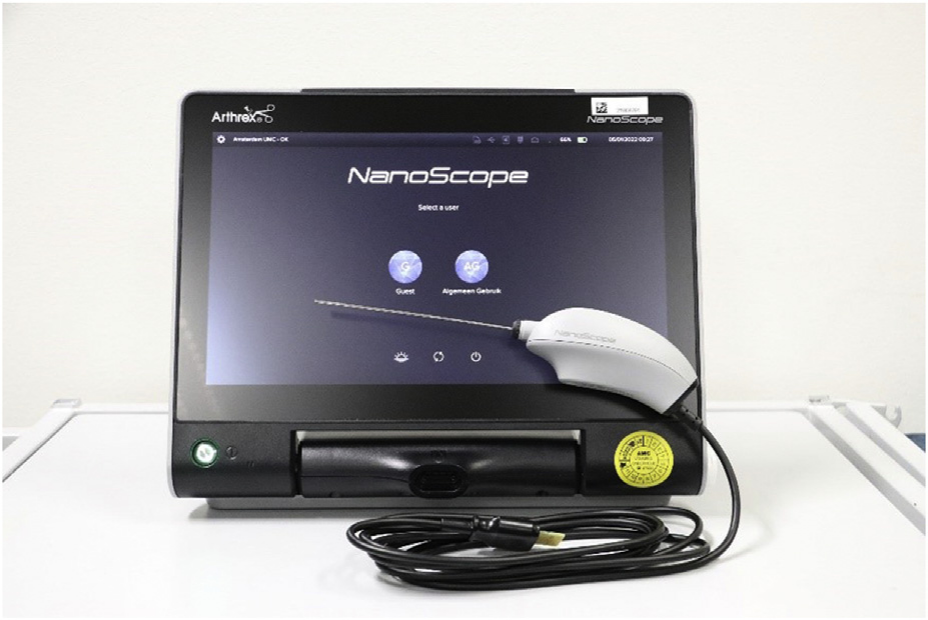
The needle arthroscopic set consists of the portable video console and the handpiece set.

The needle arthroscopic set (NanoScope, Arthrex, Naples, FL) consists of 2 parts: the sterile disposable handpiece set (Fig 1) and the portable video console (Fig 2). This handpiece set includes a semi-rigid, zero-degree needle arthroscope, sharp and blunt obturators, and corresponding sheaths. In addition, two 50-cc syringes, a 3-way tap (for connection of the syringes to the cannulas), a disposable 11-blade, and sterile sodium chloride 0.9% are used. See Table 1 for the full equipment list.

**Table 1:** Equipment List

Sterilization	Anesthesia	Needle Arthroscopy	Wound
Alcohol/chlorhexidine/iodine solution	Lidocaine 2% (10 cc)	Needle arthroscopy handpiece set	Sterile wound closure strips
Sterile draping	Red suction needle	Needle arthroscopy portable console	Compression bandage
Sterile gloves	Injection needle	Disposable 11-blade	
Cellulose mats	Syringe 10 cc	Two 50cc syringes Three-way tap Sterile sodium chloride 0.9% Pressure infuser bag Sterile collection basket Culture tubes for synovial fluid 21G (green) injection needle Arthroscopic biters	

The surgical field is disinfected and covered with sterile draping. Local anesthesia can be used in all joints (i.e., shoulder, elbow, wrist, knee, and ankle). In a sterile fashion, at least 10 cc of lidocaine 2.0% is injected along with the inflow and outflow portal tracts from the skin to the joint capsule and intra-articular. Close attention should be paid to proper anesthesia of the joint capsule, as this is well innervated.

#### Arthroscope Introduction

**Fig 3:**
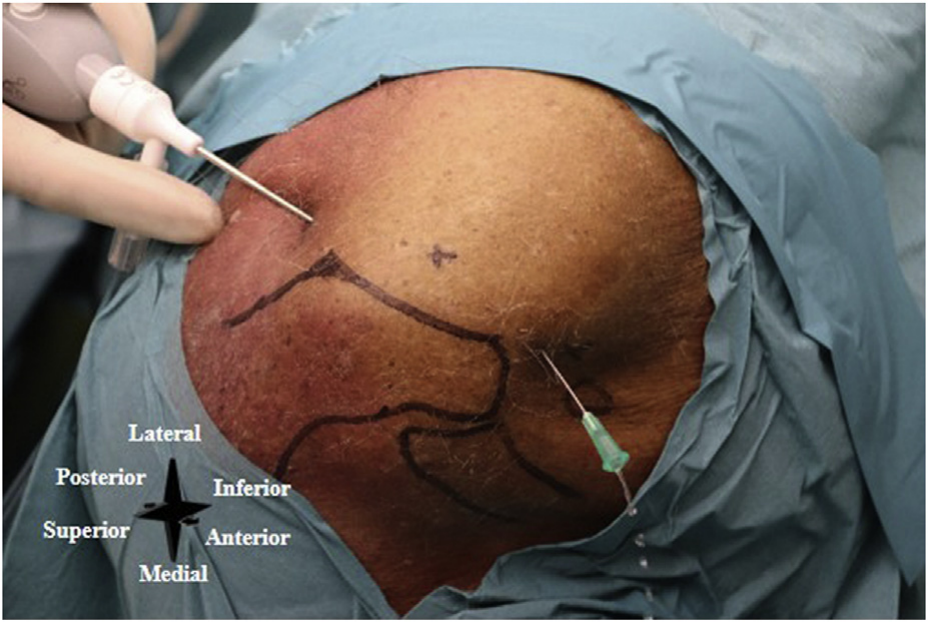
Left shoulder. A simple 21G (green) needle is used to ensure the correct outflow portal placement.

First, the desired location for the inflow portal is identified and a 2.2-mm skin incision is made with an 11-blade. Second, a 2.2-mm diameter cannula is loaded with a blunt obturator, and this cannula is then percutaneously inserted into the joint space through the stab incision. The obturator is removed, and a syringe is connected to the cannula. This syringe is used to aspirate adequate diagnostic synovial fluid for culture. Subsequently, the 1.9-mm diameter needle-arthroscope is inserted through the cannula and into the joint. Either a 50-cc syringe or a 500-cc NaCl container coupled with a pressure infuser bag is connected to the 3-way tap—which is then connected to the cannula—and the joint is distended with sterile saline. A standard arthroscopic pump can be used as well if available but is not required and provides little incremental benefit for lavage. The optional outflow portal is made under intra-articular visualization with the needle arthroscope, and a simple 21G (green) needle can be used to ensure correct portal placement (Fig 3). Further steps in the placement of the outflow portal are equal to those described previously, again using a 2.2-mm stab incision of the skin and blunt penetration of the joint capsule.

#### Lavage

As stated previously, either a 50-cc syringe or a 500-cc NaCl container coupled with a pressure infuser bag is connected to the inflow cannula. A sterile collection basket is placed under the outflow cannula (see Fig 6), and lavage is started. Lavage continues with refilled syringes or new infuser bags until it is completed and clear joint space with no remaining locations of pus or debris is observed. Once adequate positioning of the cannulas is confirmed, the needle arthroscope can be removed from the cannula. This results in less obstruction of the inflow cannula and increases the flow of saline into the joint. The inflow and outflow portal should be used interchangeably (i.e., the needle arthroscope switches to the outflow portal), and the cannulas should be repositioned throughout the entire joint, in order to sufficiently address all areas within the joint space with the flow of saline.

### Biopsies

**Fig 4:**
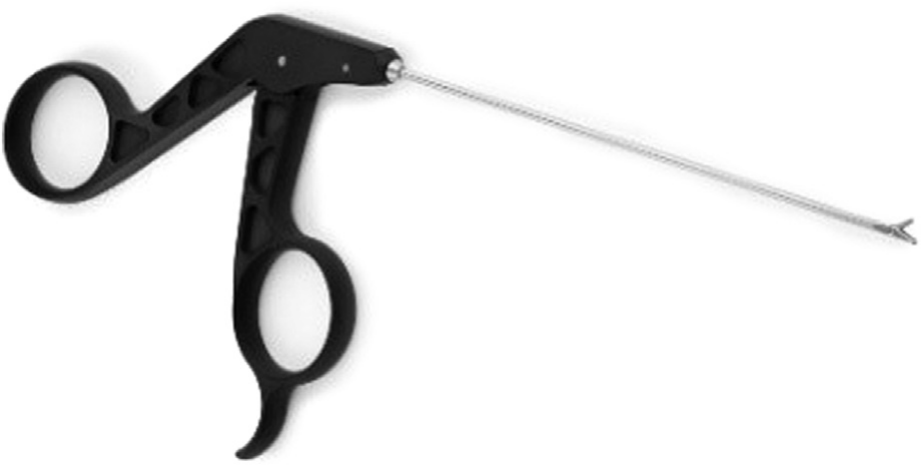
The 2-mm diameter needle arthroscopic biter/grasper.

Guided biopsies of synovial tissue can be harvested with 2-mm diameter needle arthroscopic biters (NanoBiter, Arthrex) (Fig 4). The cannula has to be removed as the arthroscopic biters do not fit through the cannula, and they can be introduced directly into the joint through the portals.

#### Closure

Sutures are not required to close the percutaneous portals. Sterile wound closure strips and a simple compression bandage for 3 days are sufficient for skin closure. Postoperative antibiotics are started according to local protocol.

### Bacterial Arthritis of a Native Shoulder

#### Patient Setup and Anatomical Landmarks

**Fig 5:**
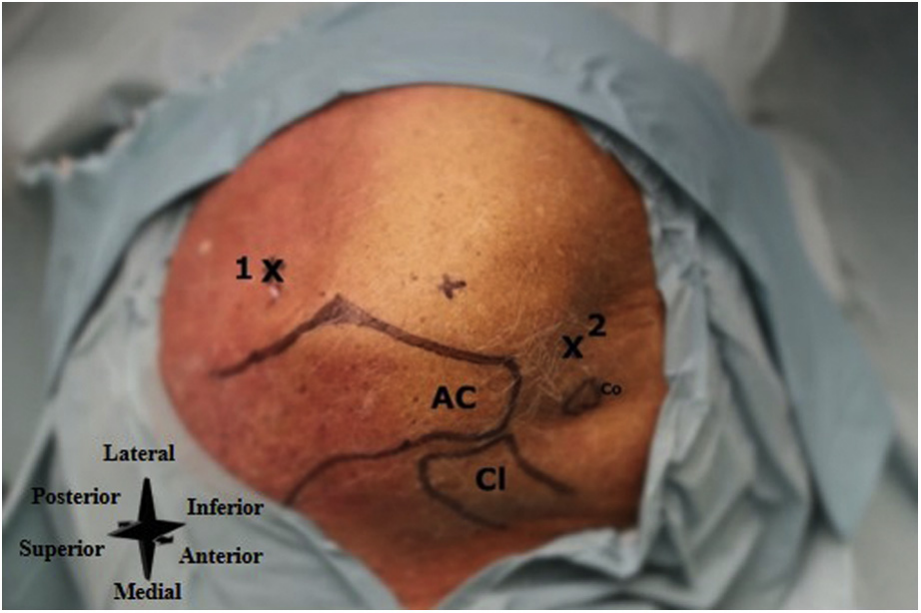
Left shoulder, seen from a superolateral perspective. Number 1 denotes the posterior portal (i.e., primary inflow portal), and number 2 the anterior portal (i.e., primary outflow portal). (AC, the acromion; Cl, clavicle; Co, coracoid process.)

The patient is placed in either a seated or lateral decubitus position with sufficient room to allow easy accessibility to the inflow and outflow portals. Anatomical landmarks (i.e., acromion, clavicle, and coracoid process) are identified and marked (Fig 5).

#### Inflow and Outflow Portal

**Fig 6:**
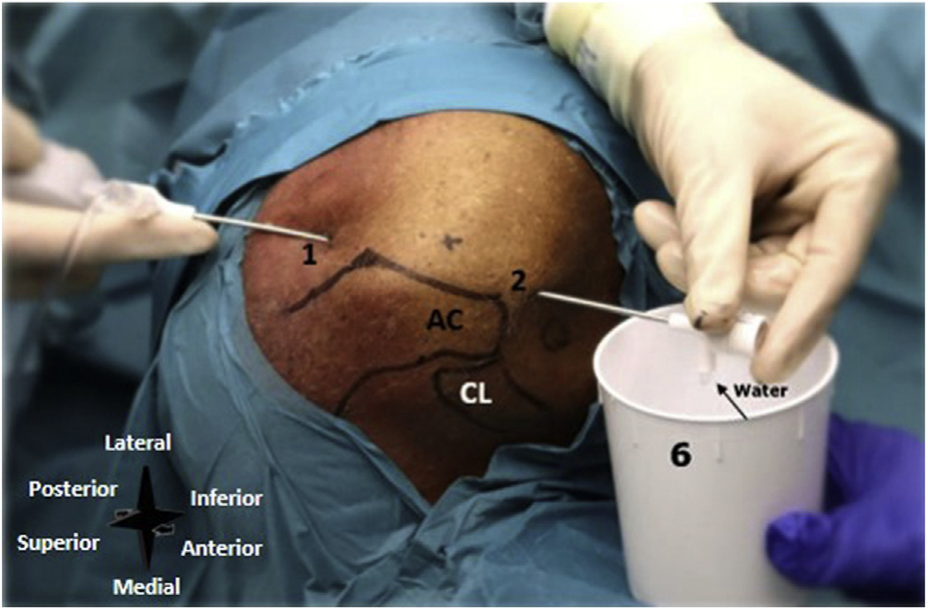
Left shoulder, seen from a superolateral perspective. The inflow and outflow portals are created. Number 1 denotes the posterior portal, number 2 the anterior portal, and number 6 the sterile basket placed to the outflow portal. (AC, the acromion; CL, clavicle.)

A posterior portal is used as the primary inflow portal and is located 2 cm inferior and 1 cm medial to the posterolateral corner of the acromion (Fig 6). An anterior portal is used as the primary outflow portal and is located lateral to the coracoid process and anterior to the acromioclavicular joint (Fig 6).

### Bacterial Arthritis of a Native Elbow

#### Patient Setup and Anatomical Landmarks

**Fig 7:**
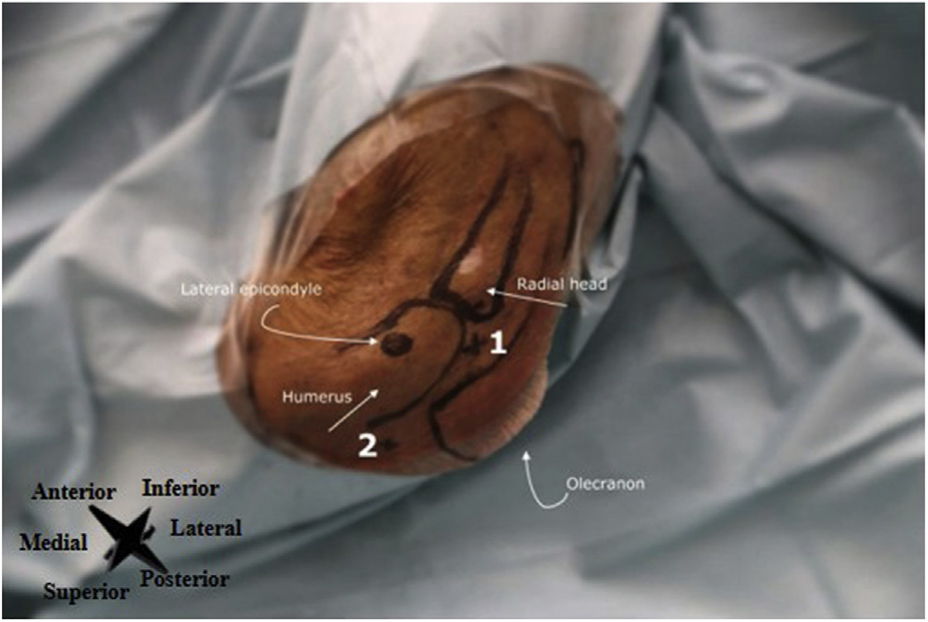
Left elbow, seen from a lateral perspective. The lateral epicondyle, radial head, olecranon, and humeral shaft are marked. Number 1 denotes the direct lateral portal (i.e., primary inflow portal), and number 2 the transtriceps/straight posterior portal (i.e., the outflow portal).

The patient is positioned lying supine with the hand of the affected side on the abdomen, the elbow in slight flexion, and with sufficient room to allow easy accessibility to the inflow and outflow portals. Anatomical landmarks for arthroscopic lavage and portal placement are the lateral epicondyle of the humerus, olecranon, and radial head, which are identified and marked (Fig 7).

#### Inflow and Outflow Portal

**Fig 8:**
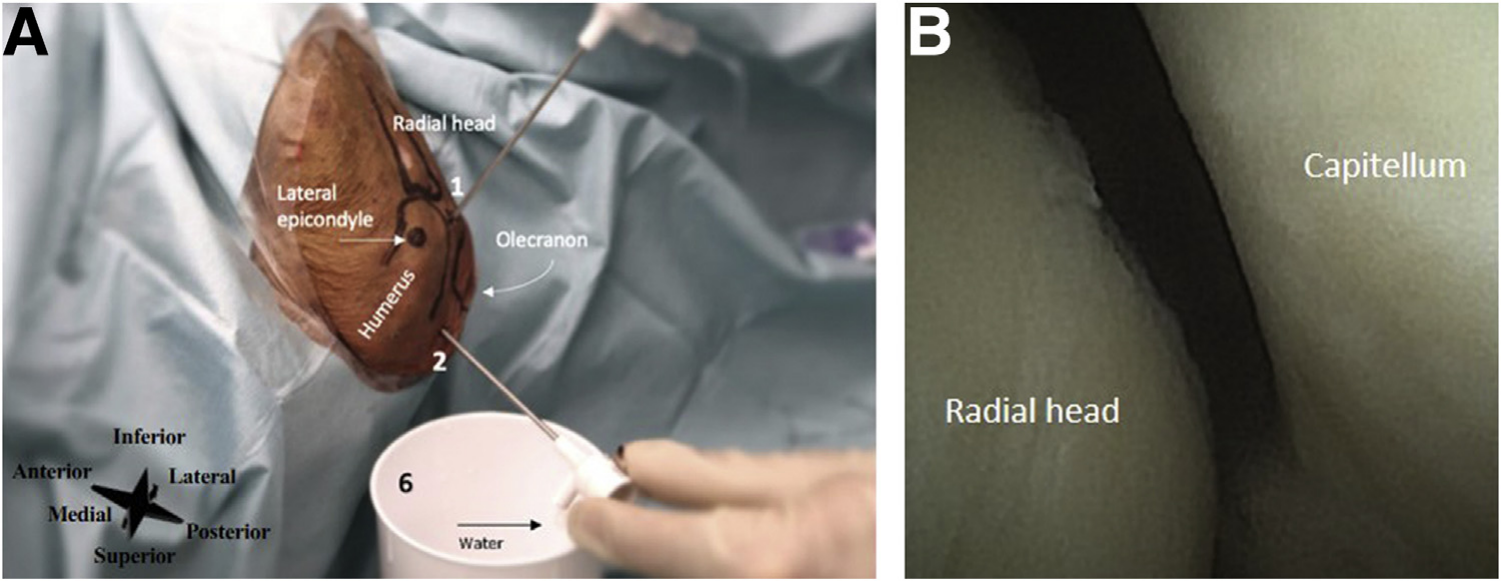
(A) Left elbow, seen from a lateral perspective. The inflow and outflow portals are created. The lateral epicondyle, radial head, olecranon, and humerus are marked. Number 1 denotes the direct lateral portal (i.e., primary inflow portal), number 2 the transtriceps/straight posterior portal (i.e., the primary outflow portal), and number 6 the sterile basket placed to the outflow portal. (B) Intra-articular needle arthroscopic view of the left elbow.

The direct lateral portal is used as the primary inflow portal and is located at the soft spot in the triangle formed by the olecranon, radial head, and lateral epicondyle (Fig 8). The transtriceps straight posterior portal is used as the primary outflow portal and is located 3 cm proximal to the olecranon in the midline through the triceps (Fig 8A). See Figure 8B for an intra-articular needle arthroscopic view of the elbow.

### Bacterial Arthritis of a Native Wrist

#### Patient Setup and Anatomical Landmarks

**Fig 9:**
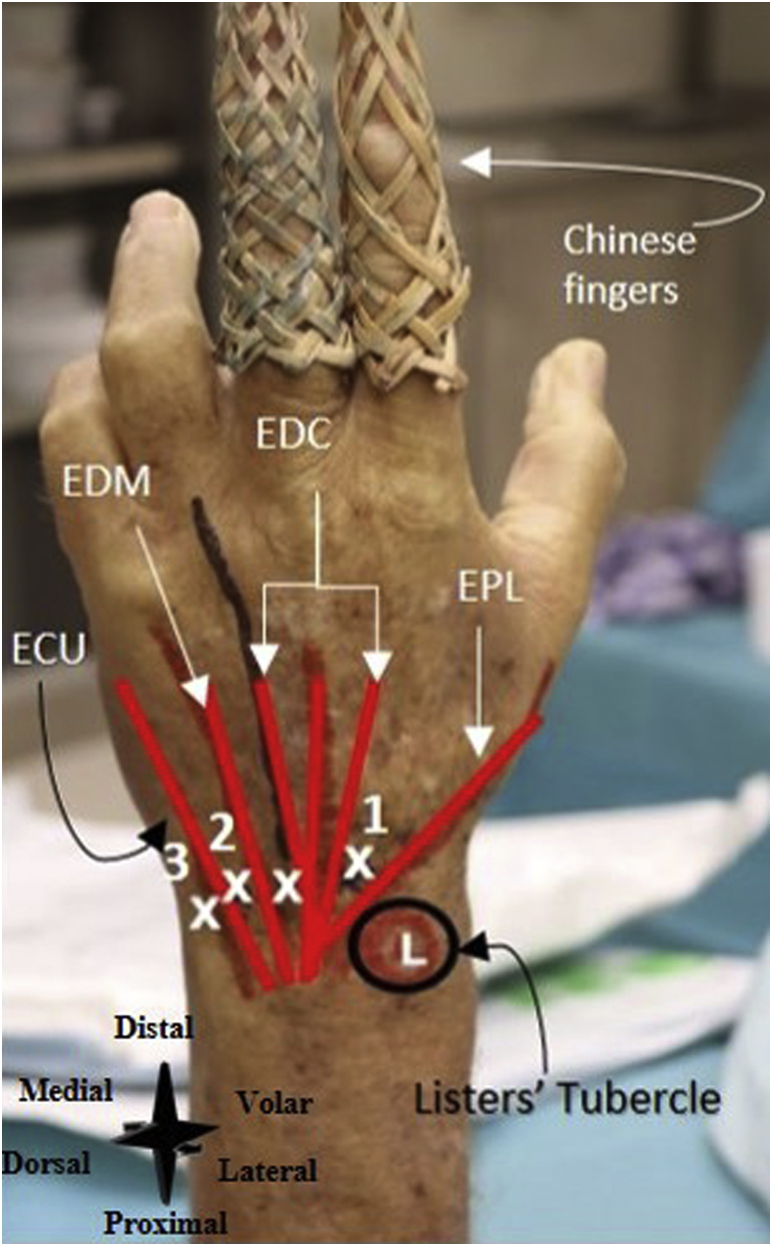
Left wrist, seen from a dorsal perspective. Two Chinese finger traps are used for the distraction of the wrist. Number 1denotes the 3-4 portal (i.e., the primary inflow portal), number 2 the 6R-portal (i.e., the primary outflow portal), and 3 the 6U-portal (the secondary outflow portal). (ECU, tendon of extensor carpi ulnaris; EDC, tendon of extensor digitorum communis muscle; EDM, tendon of extensor digiti minimi muscle; EPL, tendon of the extensor pollicis longus muscle; L, Lister's tubercle.)

The patient is positioned lying supine with the hand connected to Chinese finger traps (Fig 9). An assistant or infusion pole may help in applying distraction to the wrist. Anatomical landmarks (i.e., Lister tubercle, tendon of the extensor pollicis longus muscle, tendon of the extensor digitorum communis muscle, tendon of the extensor digiti minimi muscle, and tendon of the extensor carpi ulnari) are identified and marked (Fig 9).

#### Inflow and Outflow Portal

**Fig 10:**
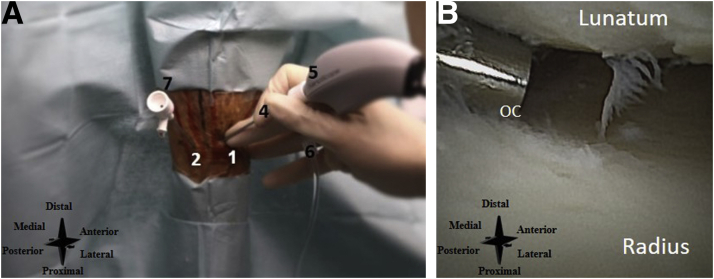
(A) Left wrist, seen from a dorsal perspective. The inflow and outflow portals are created. Number 1 denotes the primary inflow portal, 2 the primary outflow portal, 3, the secondary outflow portal, 4 the inflow cannula, 5 the needle arthroscopy camera, 6 the connected infusion bag, and 7 the outflow cannula. (B) Intra-articular needle arthroscopic view of the left wrist. OC denotes the outflow cannula.

The 3-4 portal is used as the primary inflow portal and is located 1 cm distal to Lister's tubercle, between the tendons of EPL and EDC (Fig 10A). The 6R-portal is used as the primary outflow portal and the 6U-portal is used as the secondary outflow portal. They are located radial to the tendon of ECU and ulnar to the tendon of ECU, respectively (Fig 10A). See Figure 10B for an intra-articular needle arthroscopic view of the wrist.

### Bacterial Arthritis of a Native Knee

#### Patient Setup and Anatomical Landmarks

**Fig 11:**
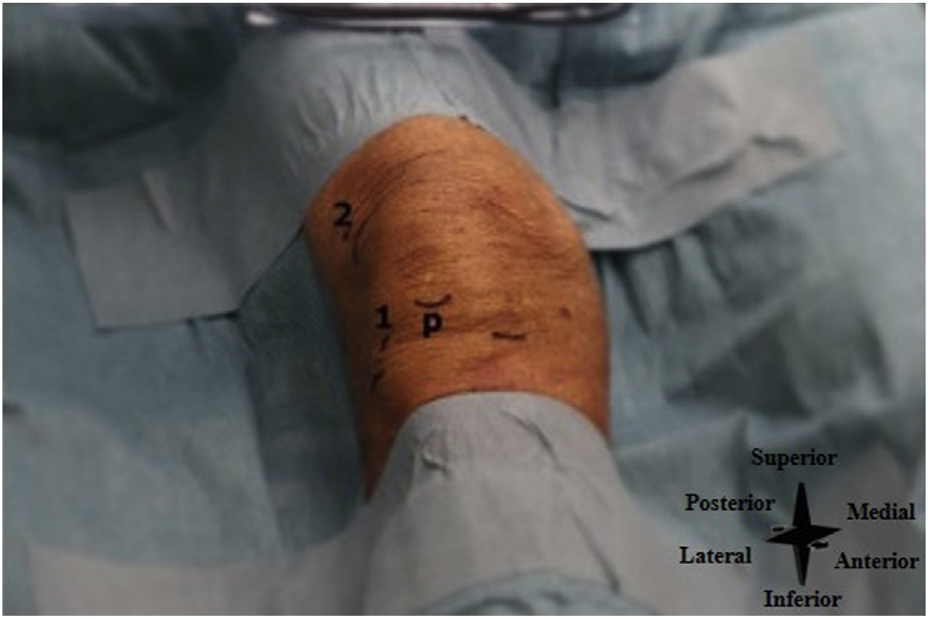
Right knee, seen from an anterior perspective. P denotes the distal part of the patella, number 1 the lateral inferior portal (i.e., the primary inflow portal), and 2 the superolateral portal (i.e., the primary outflow portal).

The patient is positioned lying supine with the knee in extension (45° of flexion is also possible), with sufficient room to allow easy accessibility to the inflow and outflow portals. Anatomical landmarks (i.e., patella, patellar tendon, and lateral joint line) are identified and marked (Fig 11).

#### Inflow and Outflow Portal

**Fig 12:**
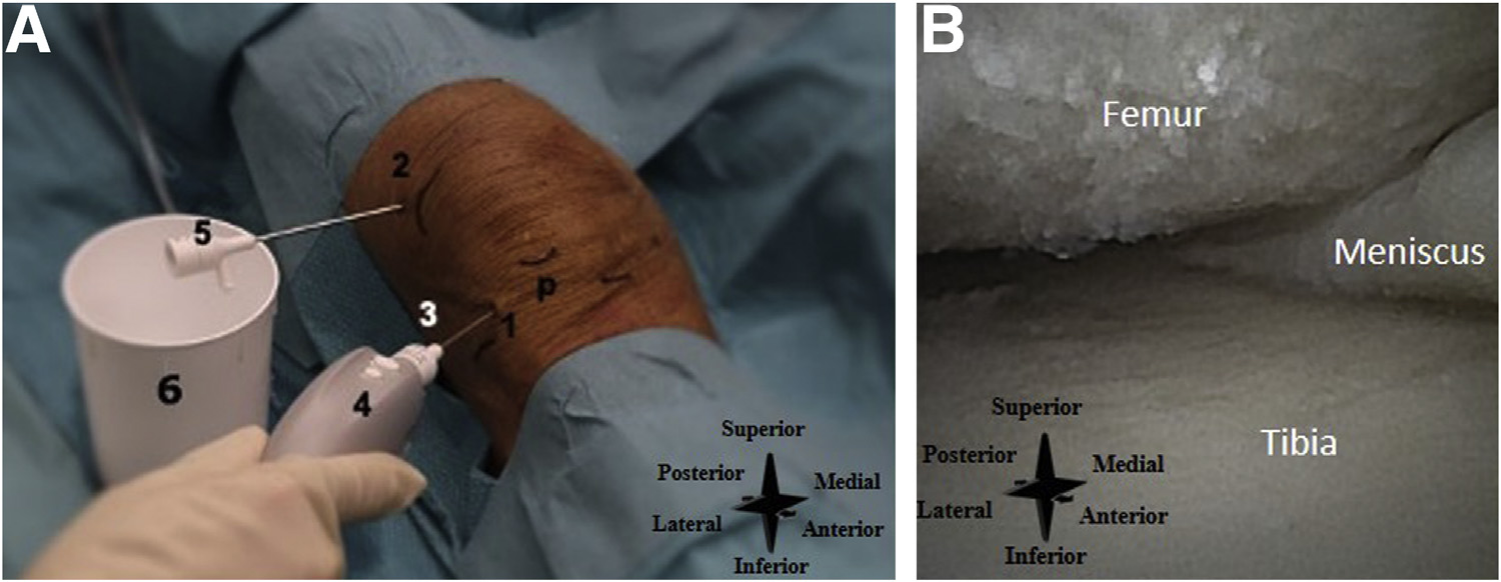
(A) Right knee, seen from an anterolateral perspective. The inflow and outflow systems are created. P denotes the distal part of the patella, number 1 the lateral inferior portal (i.e., the primary inflow portal), 2 the superolateral portal (i.e. the primary outflow portal), 3 the inflow cannula, 4 the needle arthroscopy camera, 5 the outflow cannula, and 6 the sterile basket placed under the outflow portal. (B) Intra-articular needle arthroscopic view of the right knee.

The lateral inferior portal is used as the primary inflow portal and is located one centimeter proximal to the tibial joint line and one centimeter lateral to the lateral border of the patellar tendon (Fig 12A). The superolateral portal is used as the primary outflow portal and is located at the superior aspect of the patella and one centimeter lateral to the border of the patella (Fig 12A). See Figure 12B for an intra-articular needle arthroscopic view of the knee.

### Bacterial Arthritis of a Native Ankle

#### Patient Setup and Anatomical Landmarks

**Fig 13:**
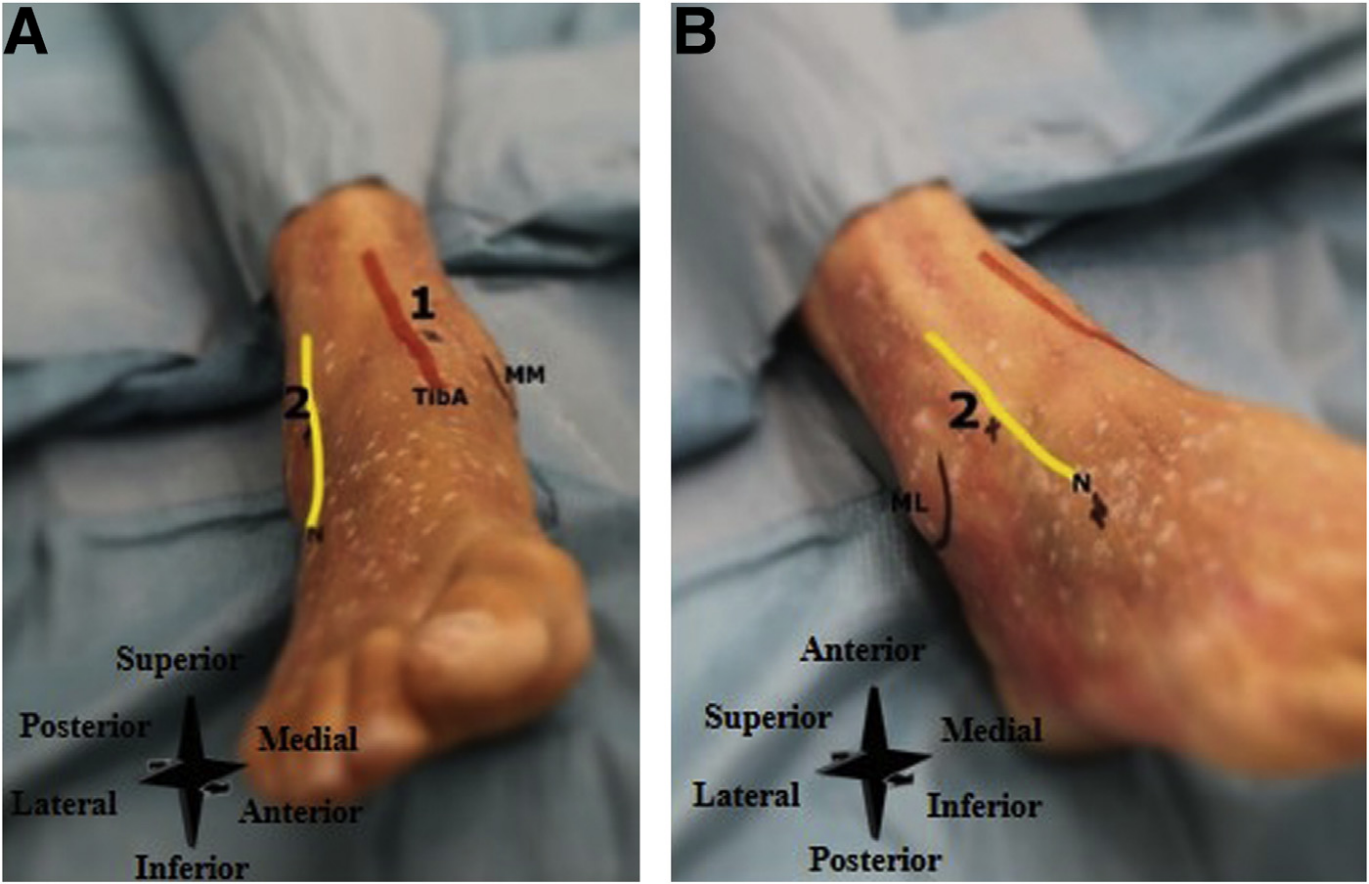
(A) Right ankle, seen from an anterior perspective. Number 1 denotes the anteromedial portal (i.e. inflow portal), and number 2 the anterolateral portal (i.e., outflow portal). (B) Right ankle, seen from an anterolateral perspective, Number 2 denotes the anterolateral portal (i.e., outflow portal). (ML, lateral malleolus; MM, medial malleolus; N, superficial peroneal nerve; TibA, tendon of the tibialis anterior muscle.)

The patient is positioned lying supine, with sufficient room to allow easy accessibility to the inflow and outflow portals. Anatomical landmarks (i.e., medial malleolus, lateral malleolus, tendon of the tibialis anterior muscle, peroneus tertius tendon, and the superficial peroneal nerve are identified and marked (Fig 13 A and B). The best method to identify the superficial peroneal nerve is the forced ankle plantar flexion and inversion. In this position, the nerve can be identified in 60% of the ankles by palpation.^4^

#### Inflow and Outflow Portal

**Fig 14:**
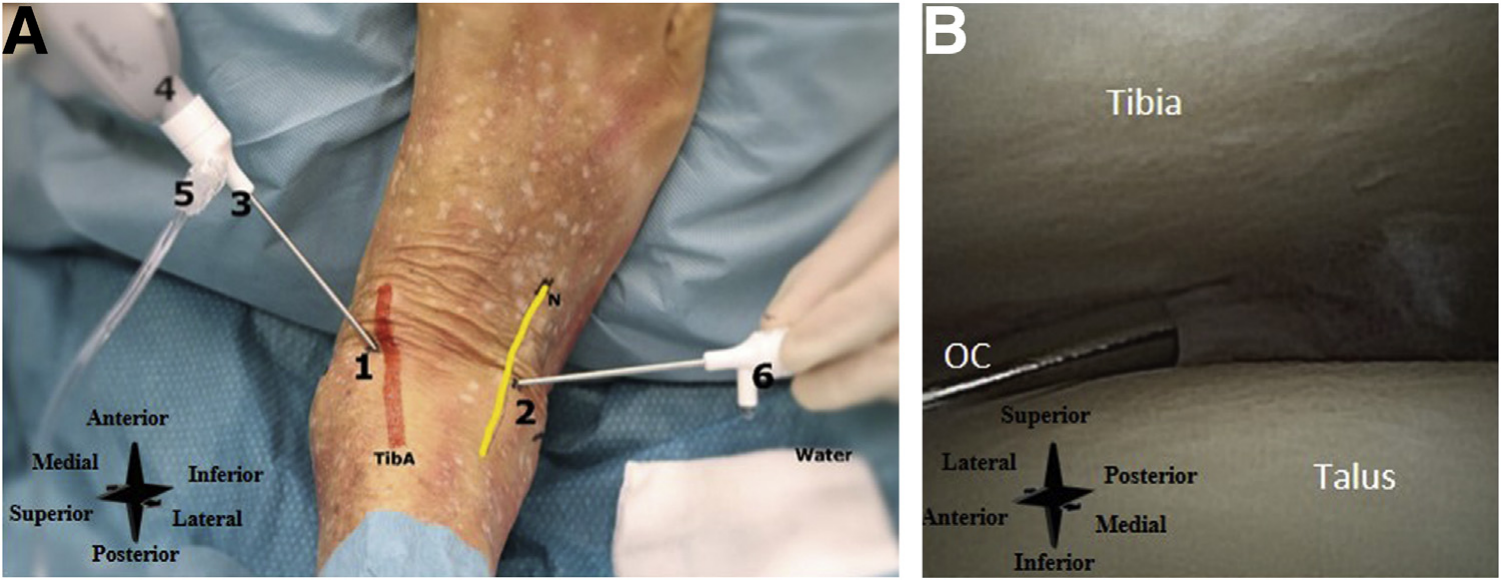
(A) Right ankle, seen from a proximal perspective, with the foot resting on the surgeon's abdomen. Number 1 denotes the inflow portal, 2 the outflow portal, 3 the cannula, 4 the needle arthroscope, 5 the connected infusion bag, and 6 the outflow cannula with water/saline coming out. (B) Intra-articular needle arthroscopic view of the right ankle. OC denotes the outflow cannula. (N, superficial peroneal nerve; TibA, tendon of the tibialis anterior muscle.)

The anteromedial portal is used as the primary inflow portal and is located on the soft spot just medial to the anterior tibial tendon and at the anterior joint line (Fig 14A). It is performed with the joint in maximal dorsiflexion to protect the cartilage on introduction. The anterolateral portal is used as the primary outflow portal. It is created under direct visualization at the anterior joint line, located lateral to the peroneus tertius tendon (or to the extensor digitorum longus in case of absence of the tertius) and superficial peroneal nerve, and medial to the lateral malleolus (Fig 14A). Slight noninvasive distraction may aid in portal placement. See Figure 14B for an intra-articular needle arthroscopic view of the ankle.

## Discussion

The needle arthroscopic technique presented here provides a minimally invasive approach to treat bacterial arthritis of a native shoulder, elbow, wrist, knee, and ankle. In line with previous research on bedside needle arthroscopy, we observed that it is an easy technique to perform for the physician, and well-tolerated by the patient.^3^^,^^5^, ^6^, ^7^, ^8^, ^9^, ^10^

When considering this technique, it is important to appreciate the limitations of this presentation and of the technique itself. First, all photos and videos presented here were fabricated for educational purposes on cadaveric specimens. In clinical cases of bacterial arthritis, vision may be more obscured due to blood, pus, and debris than presented in the images here. Second, there are several pitfalls concerning the technique itself (Table 2), including a learning curve with the new equipment. Third, although the guided lavage may be the first step in source control, the current technique does not include a synovectomy and we therefore recommend to consider bedside needle arthroscopy as an initial step in the management process. A second, more aggressive procedure may be required at a later stage; for example, in the case of severe pathology that cannot be addressed with bedside needle arthroscopy, or in case of insufficient recovery upon needle arthroscopic lavage. This stepwise approach was evaluated in a pilot study of 10 patients, which concluded that needle arthroscopy was sufficient in all 10 cases, eliminating the need for conventional surgery for these patients.^3^

**Table 2:** Pitfalls of Needle Arthroscopy in Bacterial Arthritis

The zero-degree viewing angle, which may be unfamiliar to surgeons, has a learning curve.
The needle arthroscopic instruments are smaller and less rigid than conventional arthroscopy. This may increase difficulty and procedure time, and it has a learning curve.
If performed under local anesthesia, conversion to general or spinal anesthesia may be necessary if the procedure is not tolerated by the patient.
If performed under local anesthesia, close attention should be paid to proper anesthesia of the joint capsule, as this is well innervated
The flow of saline through the needle arthroscopic cannula is lower compared with conventional arthroscopy. This can be remedied by removing the camera from the cannula to create higher flow once the cannula is correctly positioned in the joint.
In case of difficult introduction into the joint, the sharp obturator can be used instead of the blunt obturator; however, this carries a risk of iatrogenic damage and therefore requires extra caution.

This stepwise approach has multiple advantages (Table 3), including less soft tissue damage, the possibility to perform needle arthroscopy under local anesthesia requiring less personnel and hospital facilities (e.g., operation theater/operation room time), and combining a diagnostic (adequate diagnostic joint aspiration during portal placement) and therapeutic (direct lavage following aspiration) approach. This prompt bedside treatment may prevent severe illness and start rapid recovery and altogether, needle arthroscopy may result in reduction of morbidity, hospital stay, and costs. However, larger studies should be performed to confirm these advantages and therefore a prospective observational cohort study was started at our university medical center.

**Table 3:** Advantages of Needle Arthroscopy in Bacterial Arthritis

Decreased soft-tissue damage
It can be performed under local anesthesia, which leads to less need for personnel and hospital facilities
Used as diagnostic and therapeutic procedure
Possibility to take biopsies
Expedited recovery
Potential cost-savings

In conclusion, this surgical guideline describes bedside needle arthroscopy as the initial diagnostic and therapeutic step in the management of patients with suspected bacterial arthritis of a native shoulder, elbow, wrist, knee, and ankle. It may ensure that true positive patients are addressed in a timely fashion yet decrease the burden of overtreatment for false-positive cases.

## Supplementary Data

ICMJE author disclosure forms

Video 1 The surgical technique of the needle arthroscopy for each joint. Patient setup, anatomical landmarks, and the inflow and outflow portals are clarified per joint. The surgical technique of the shoulder starts at 0:48 minutes, the elbow at 1:31 minutes , the wrist at 2:17 minutes , the knee at 2:54 minutes , and the ankle at 3:41 minutes.

Pockey Cards
