# Supplementary material for: Needle Arthroscopy for Bacterial Arthritis of a Native Joint: Surgical Technique for the Shoulder, Elbow, Wrist, Knee, and Ankle Under Local Anesthesia
Source: Arthrosc Tech. 2022 Sep 21;11(9):e1641–8. doi: 10.1016/j.eats.2022.05.011 (PMC9520080; doi:10.1016/j.eats.2022.05.011)
Supplement: Pockey Cards [file mmc3.pdf]

Amsterdam UMC
University Medical Centers

Pocket cards
April 2022

A minimally invasive approach to treatment of bacterial arthritis: needle arthroscopy

Under local anesthesia

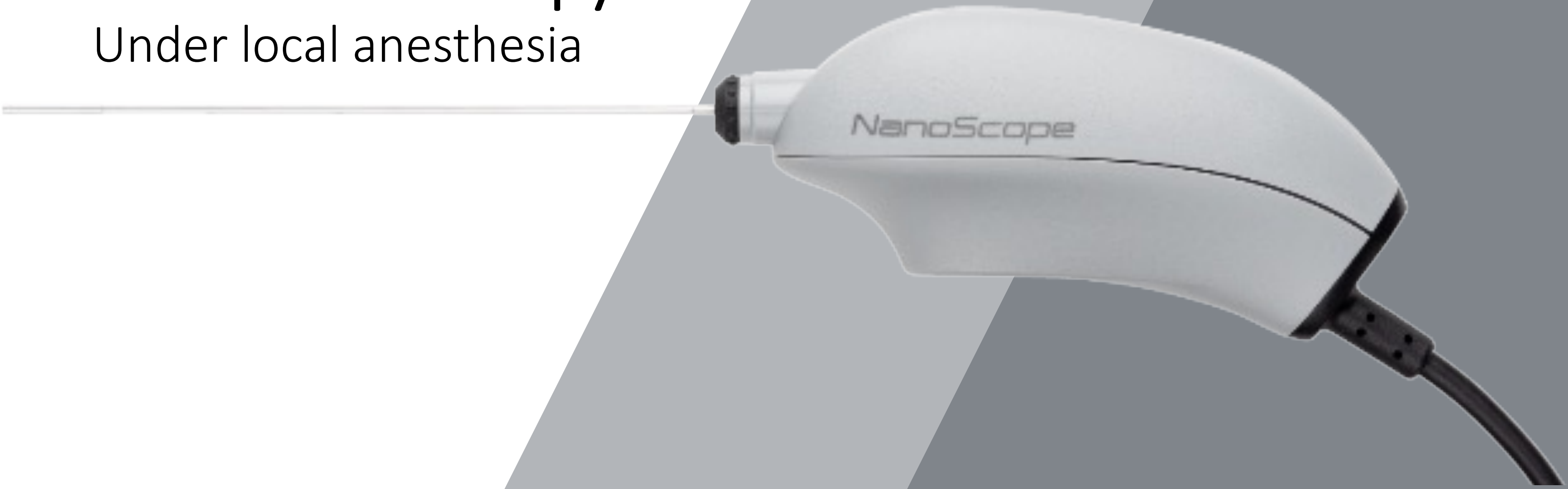

Walinga et al. 2022

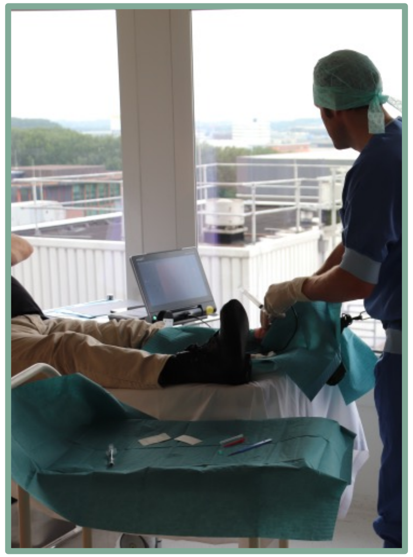

Material

Sterilization	Anesthesia	NanoScopy	Woundcare
❑ Alcohol/chlorhexidine	❑ Lidocaine 2% (10cc)	❑ Needle arthroscopy handpiece set	❑ Sterile wound closure strips
❑ Sterile draping	❑ Red aspiration needle	❑ Needle arthroscopy portable console	❑ Compression bandage
❑ Sterile gloves	❑ Injection needle	❑ Disposable 11-blade	
	❑ Syringe 10cc	❑ Two 50cc syringes	
		❑ Three-way tap	
		❑ Sterile sodium chloride 0.9%	
		❑ Pressure infuser bag	
		❑ Sterile collection basket	
		❑ Culture tubes for synovial fluid	
		❑ 21G (green) injection needle	
		❑ Arthroscopic graspers	

The disposable handpiece set (sterile)

- A = The needle arthroscope
- B = HDMI connection of the needle arthroscope to the portable console
- C1 = Blunt trocar
- C2 = Sharp trocar
- D = Cannula
- E = Calibration flag, may only be removed after the needle arthroscope is calibrated
- F = Biter/Grasper to take biopsies

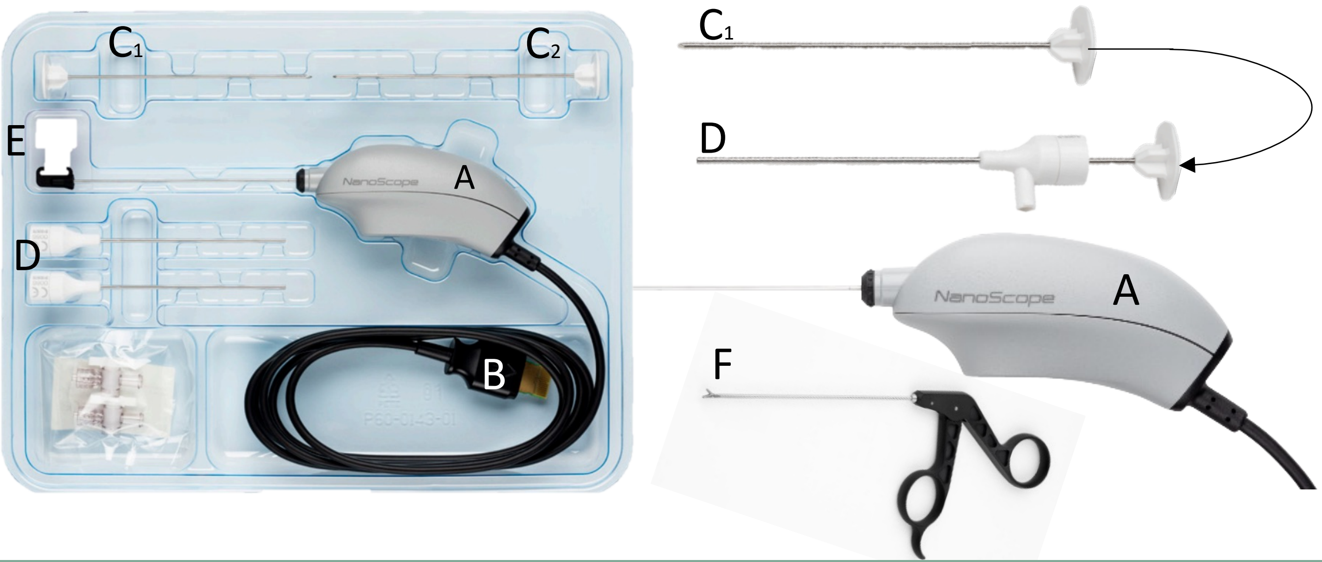

Walinga et al. 2022 Arthroscopy Techniques

The Shoulder

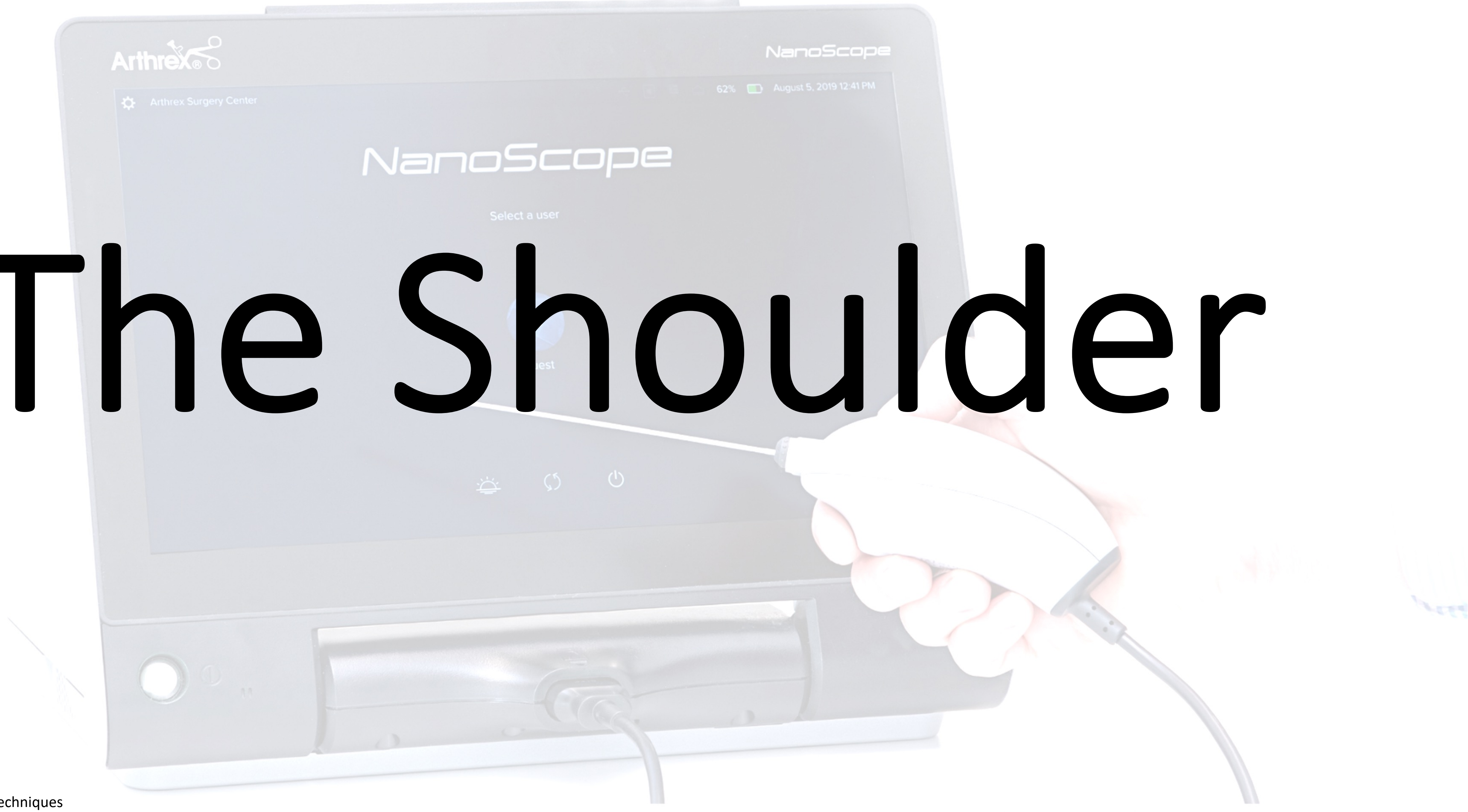

Walinga et al. 2022 Arthroscopy Techniques

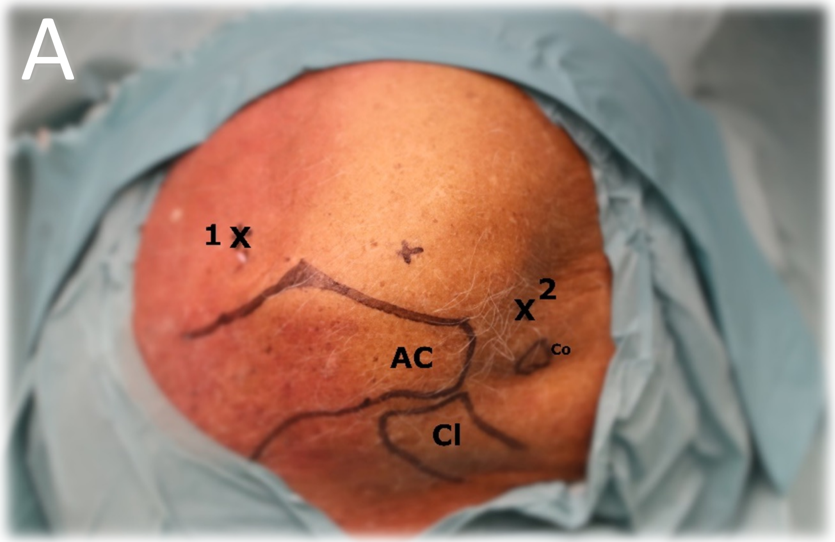

A. <u>Position</u>: the patient is positioned on the not affected side in a lateral decubitus position (sitting with a straight back is also possible). Inject local anesthesia (lidocaine 2%) to portals 1 and 2, and intra-articular. Make sure that the joint capsule is well anesthetized.

B. <u>Portals:</u>
   1) Posterior portal: 2 cm inferior and 1 cm medial to the posterolateral corner of the acromion (AC)
   2) Anterior portal: lateral to coracoid process (co) and anterior to the AC-joint (AC-Cl)

C. <u>Procedure portal</u> 1: make a small 2.2-mm incision, and insert the blunt trocar with cannula into the joint (3). Connect an empty syringe to the cannula and aspirate synovial fluid for culture. Insert the needle arthroscopy through the cannula (4). Connect a 50cc syringe or a 500cc NaCl container (5) coupled with a pressure bag to the cannula (3).

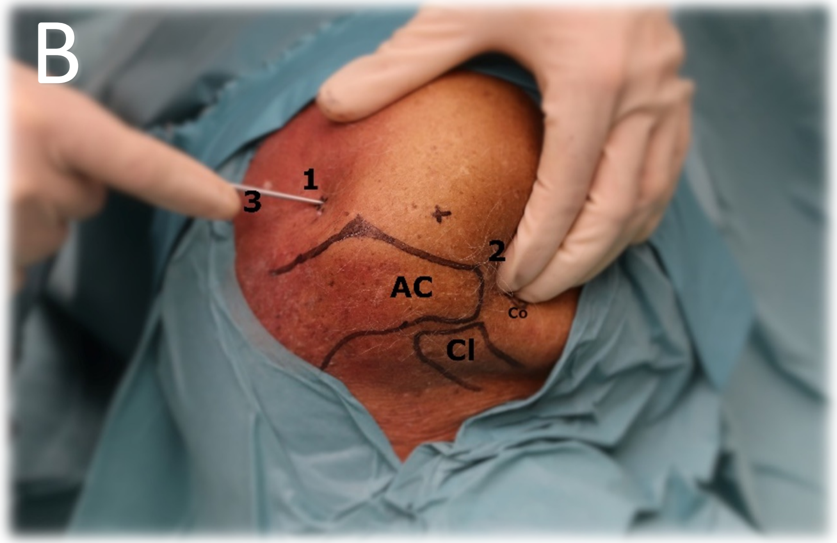

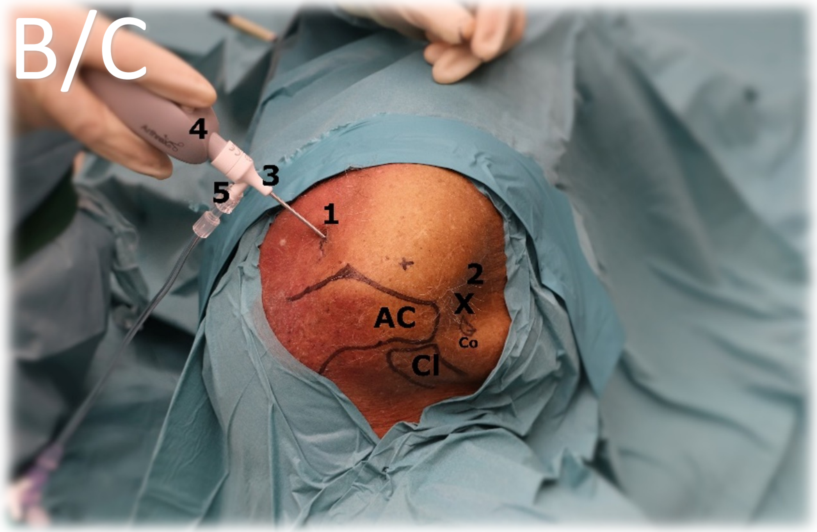

D. <u>Procedure portal</u> 2: make a small 2.2-mm incision under visualization with the needle arthroscope with a blunt trocar (6)

TIPS:
- When you remove the camera from the cannula, the flow of saline may be increased, so you can have a better lavage;
- It is possible to take biopsies for diagnostic purposes with a grasper;
- Alternative: single portal lavage (preference for portal 1);
- When it is hard to get access to the joint, you can use the sharp trocar (be careful with the humerus head).

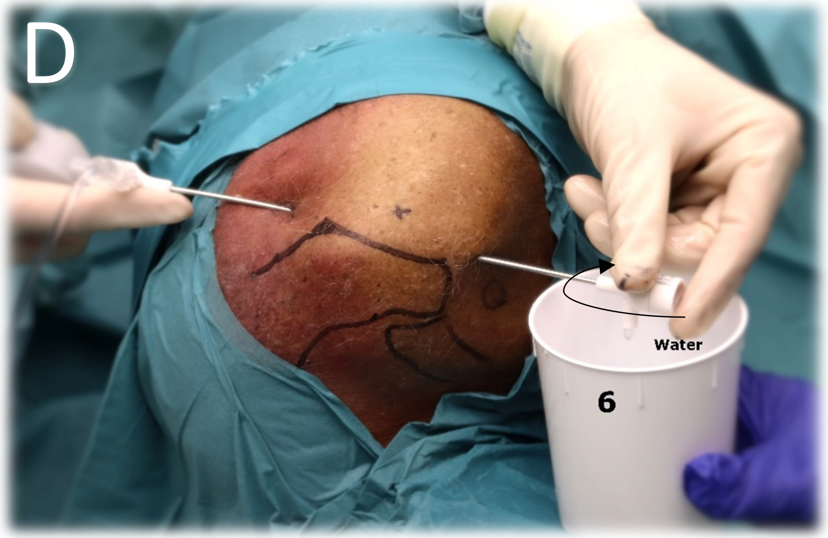

The Elbow

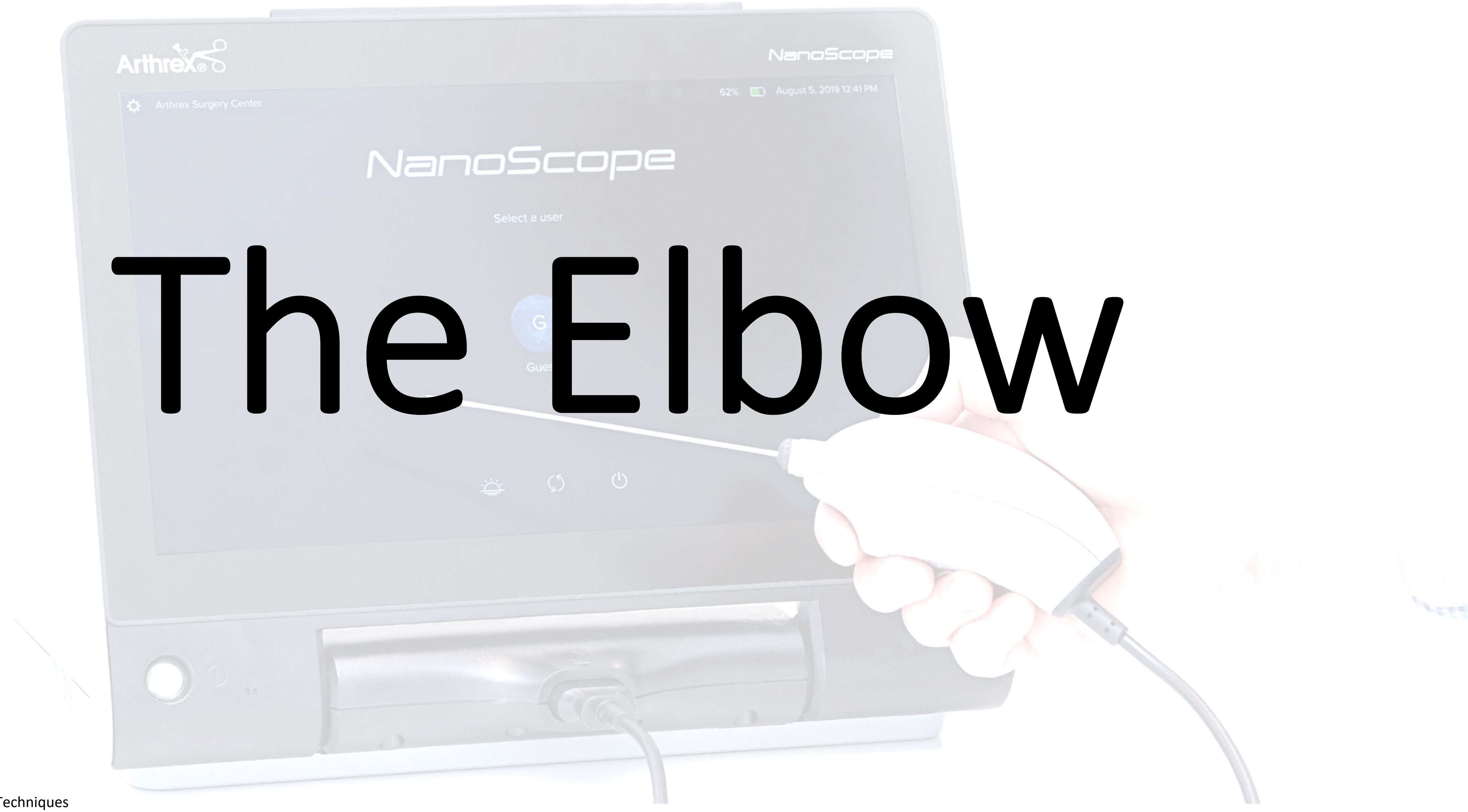

Walinga et al. 2022 Arthroscopy Techniques

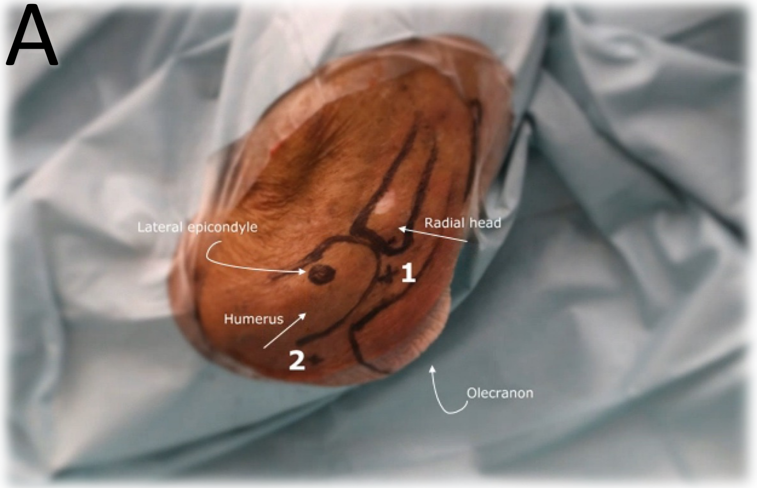

A. Position: the patient is positioned supine, with the wrist of the affected side on the abdomen, and with the elbow in slight flexion. Inject local anesthesia (lidocaine 2%) to portals 1 and 2, and intra-articular. Make sure that the joint capsule is well anesthetized.

B. Portals:
   1) Soft spot/direct lateral portal: in the triangle of the olecranon, radial head, and the lateral epicondyle;
   2) Transtriceps/straight posterior portal: 3cm proximal to the olecranon, midline of the triceps.

C. Procedure portal 1: make a small 2.2-mm incision, and insert the blunt trocar with cannula into the joint (3). Connect an empty syringe to the cannula and aspirate synovial fluid for culture. Insert the needle arthroscopy through the cannula (4). Connect a 50cc syringe or a 500cc NaCl container (5) coupled with a pressure bag to the cannula (3).

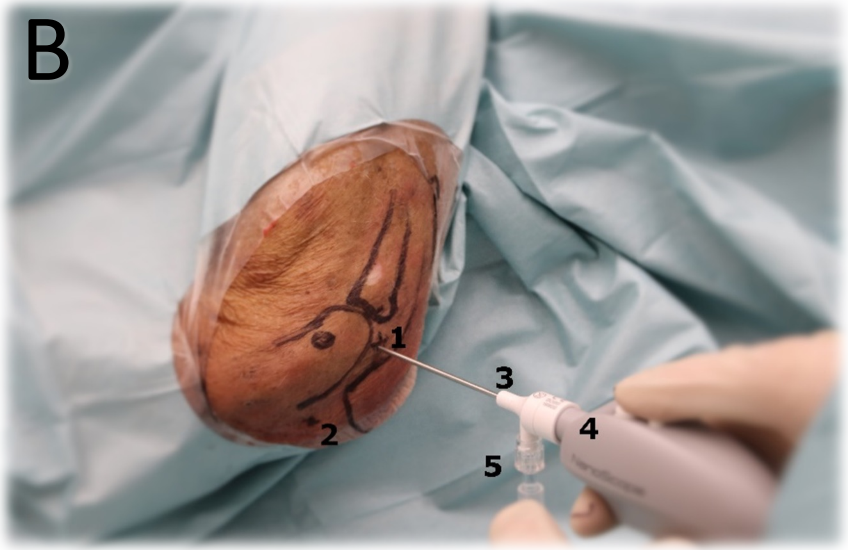

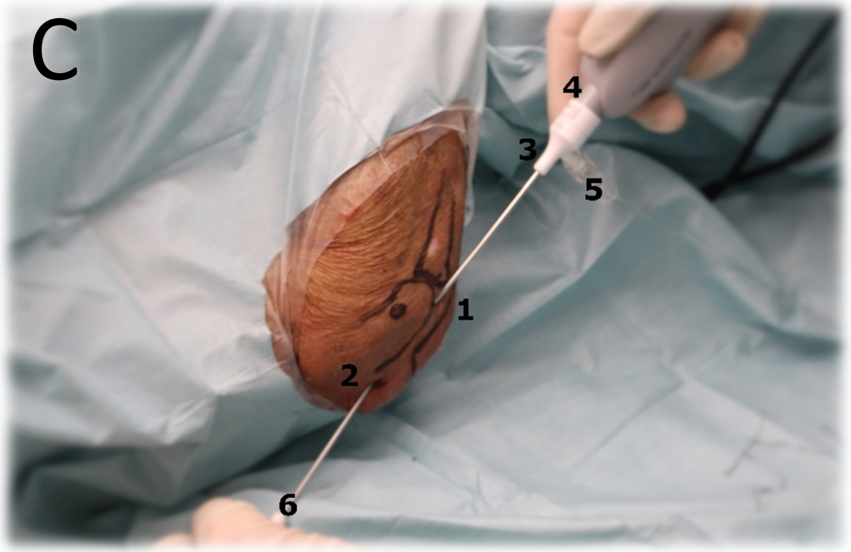

D. Procedure portal 2: make a small 2.2-mm incision under visualization with the needle arthroscope with a blunt trocar (6)

TIPS:
- When you remove the camera from the cannula, the flow of saline may be increased, so you can have a better lavage;
- It is possible to take biopsies for diagnostic purposes with a grasper;
- Alternative: single portal lavage (preference for portal 1).

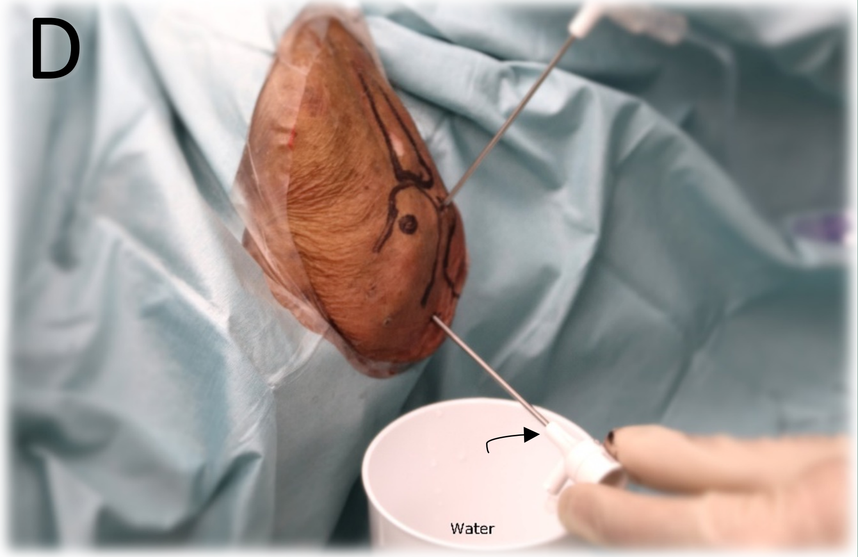

Walinga et al. 2022 Arthroscopy Techniques

The Wrist

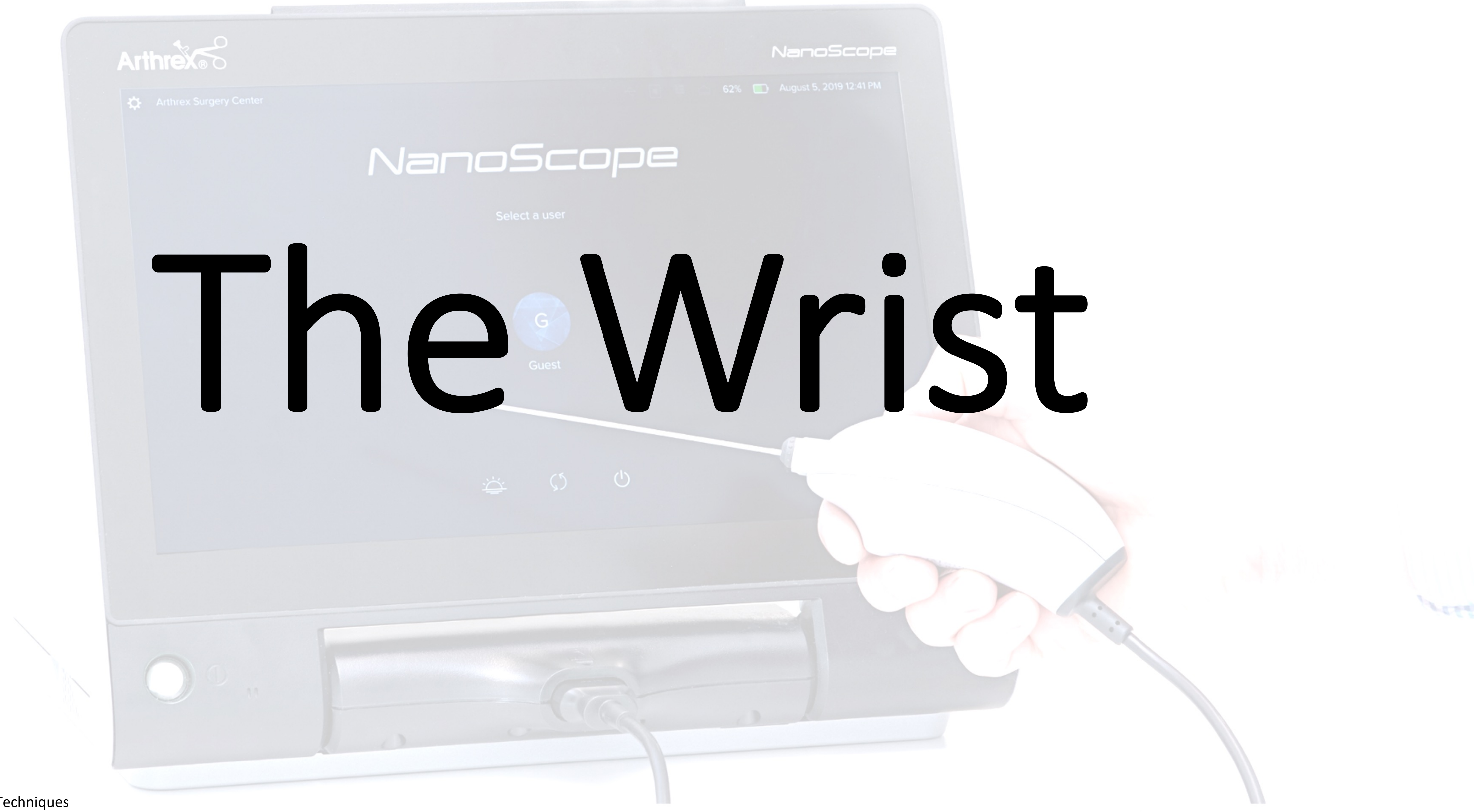

Walinga et al. 2022 Arthroscopy Techniques

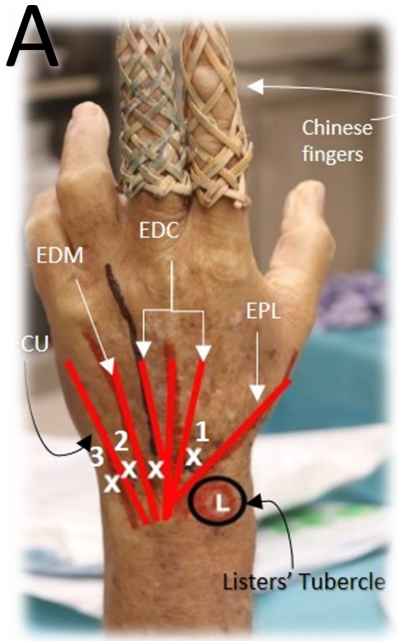

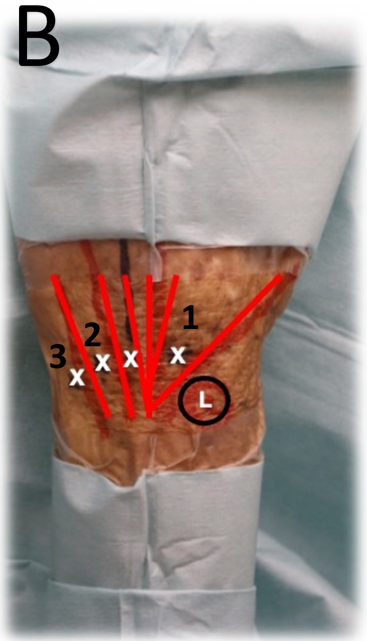

A. <u>Position</u>: the patient is positioned supine with the hand connected to Chinese finger traps. Inject local anesthesia (lidocaine 2%) to portals 1 and 2, and intra-articular. Make sure that the joint capsule is well anesthetized.

B. <u>Portals:</u>
   1) 3-4 portal: 1 centimeter distal to Lister's tubercule, between the tendons of EPL (II) and EDC (IV);
   2) 6R-portal: radial to the tendon of the ECU (VI);
   3) 6U-portal: ulnar to the tendon of the ECU (VI).

C. <u>Procedure portal</u> 1: make a small 2.2-mm incision, and insert the blunt trocar with cannula into the joint (3). Connect an empty syringe to the cannula and aspirate synovial fluid for culture. Insert the needle arthroscopy through the cannula (4). Connect a 50cc syringe or a 500cc NaCl container (5) coupled with a pressure bag to the cannula (3).

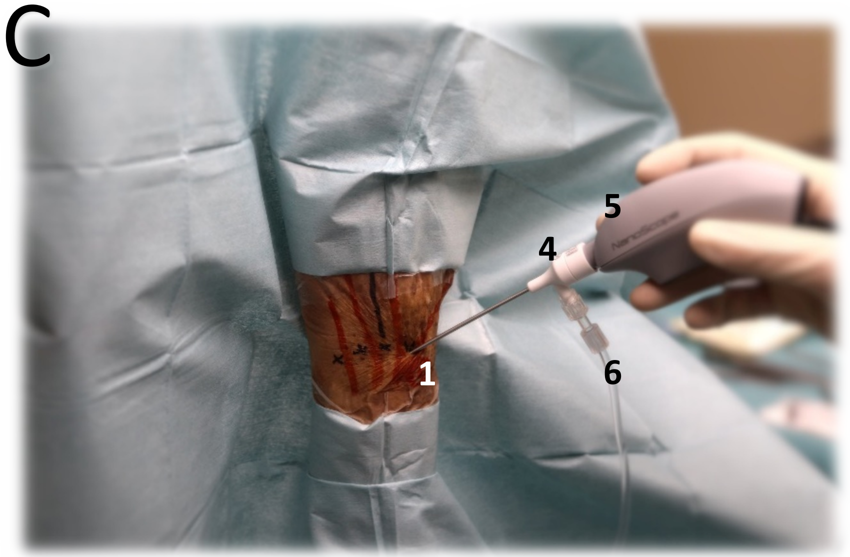

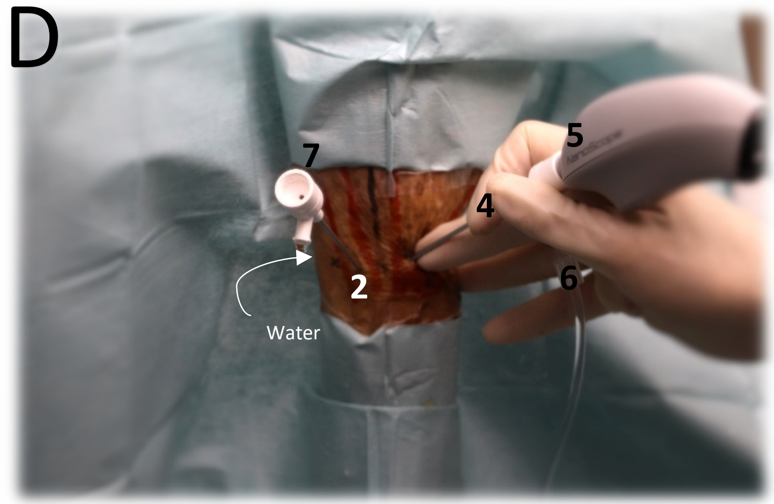

D. <u>Procedure portal 2 or 3</u>: make a small 2.2-mm incision under visualization with the needle arthroscope with a blunt trocar (6)

TIPS:
- When you remove the camera from the cannula, the flow of saline may be increased, so you can have a better lavage;
- It is possible to take biopsies for diagnostic purposes with a grasper;
- An assistant or infusion pole may help in applying distraction to the wrist
- Alternative: single portal lavage (preference for portal 1).

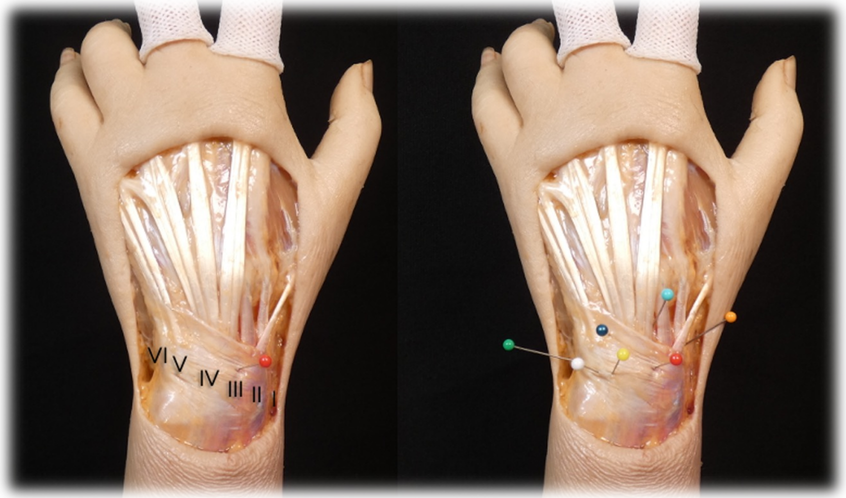

Reprinted by permission from Springer Nature Customer Service Centre GmbH: Springer Nature, Springer eBook, Wrist Portals and Arthroscopic Anatomy, F. Corella, M. Ocampos, M. C. Manzanares-Céspedes et al. Copyright 2022

The Knee

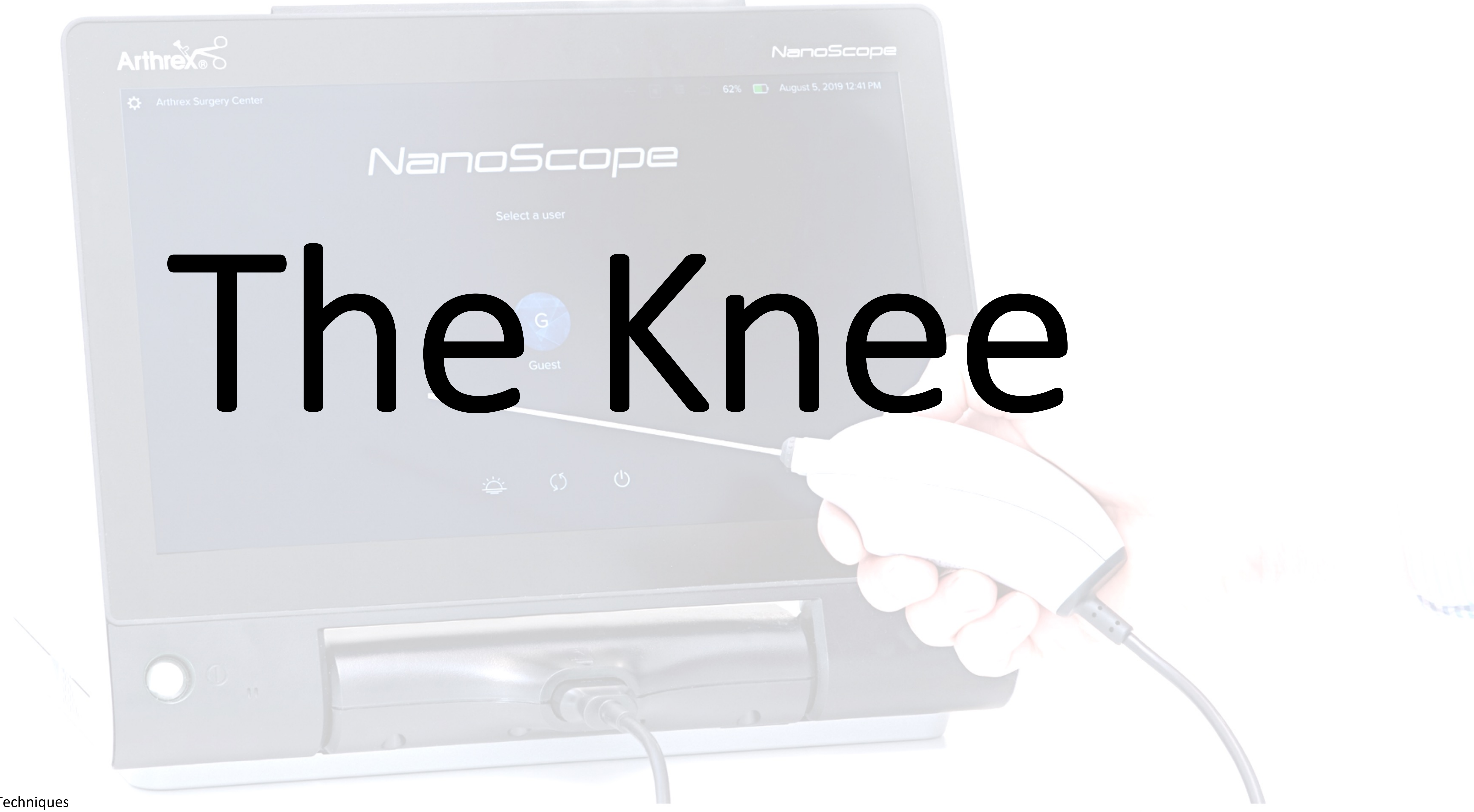

Walinga et al. 2022 Arthroscopy Techniques

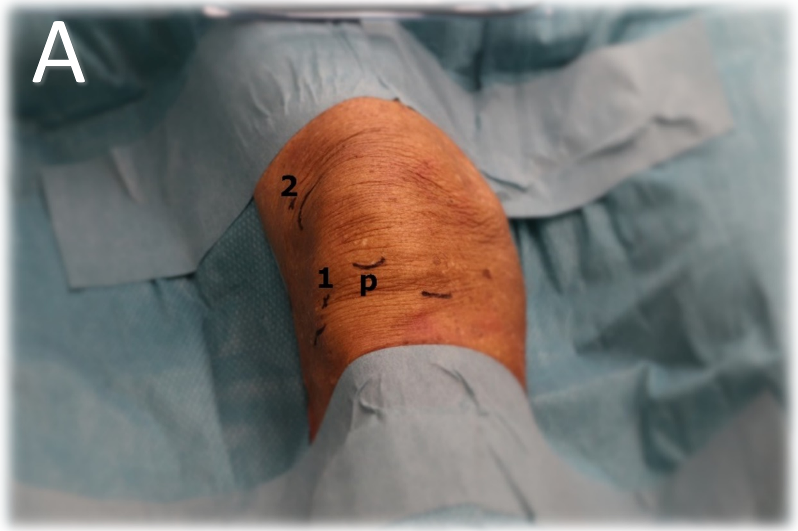

A. <u>Position</u>: the patient is positioned supine, with the knee in extension (or slight flexion). Inject local anesthesia (lidocaine 2%) to portals 1 and 2, and intra-articular. Make sure that the joint capsule is well anesthetized.

A. <u>Portals:</u>
   1) Lateral inferior portal: 1 cm above the tibial joint line and 1 cm lateral to the border of the patellar tendon
   2) Superolateral portal: superior aspect of the patella and 1 cm lateral to the lateral border of the patella

B. <u>Procedure portal</u> 1: make a small 2.2-mm incision, and insert the blunt trocar with cannula into the joint (3). Connect an empty syringe to the cannula and aspirate synovial fluid for culture. Insert the needle arthroscopy through the cannula (3). Connect a 50cc syringe or a 500cc NaCl container (4) coupled with a pressure bag to the cannula (3).

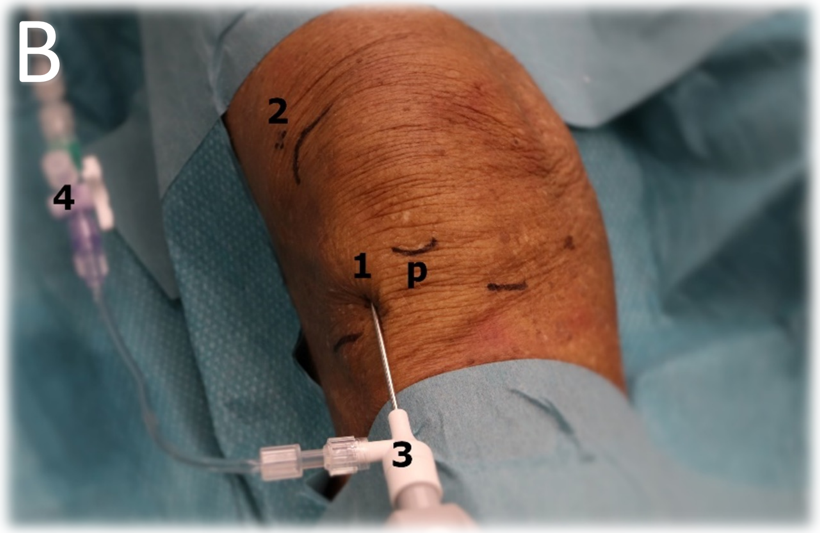

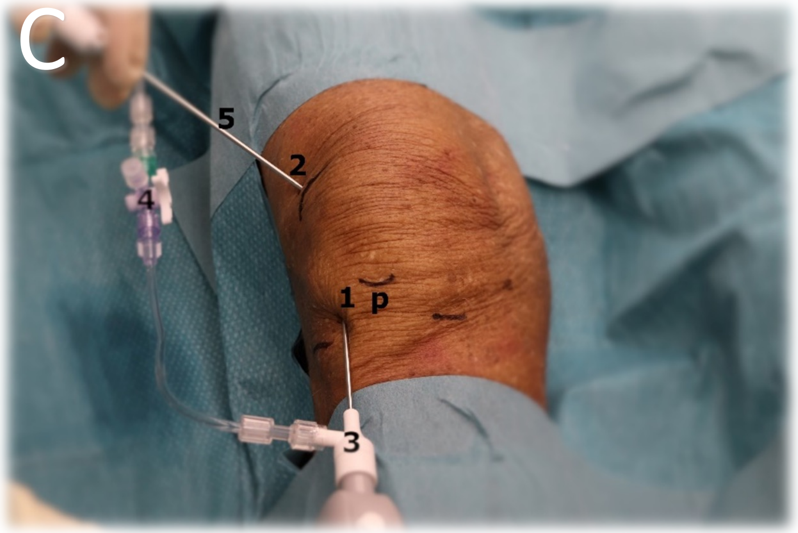

D. <u>Procedure portal</u> 2: make a small 2.2-mm incision under visualization with the needle arthroscope with a blunt trocar (6)

TIPS:
- When you remove the camera from the cannula, the flow of saline may be increased, so you can have a better lavage;
- It is possible to take biopsies for diagnostic purposes with a grasper;
- Knee in hyperextension gives easier access along with the patella to portal 1
- A medial portal can be made under visualization of the needle arthroscope

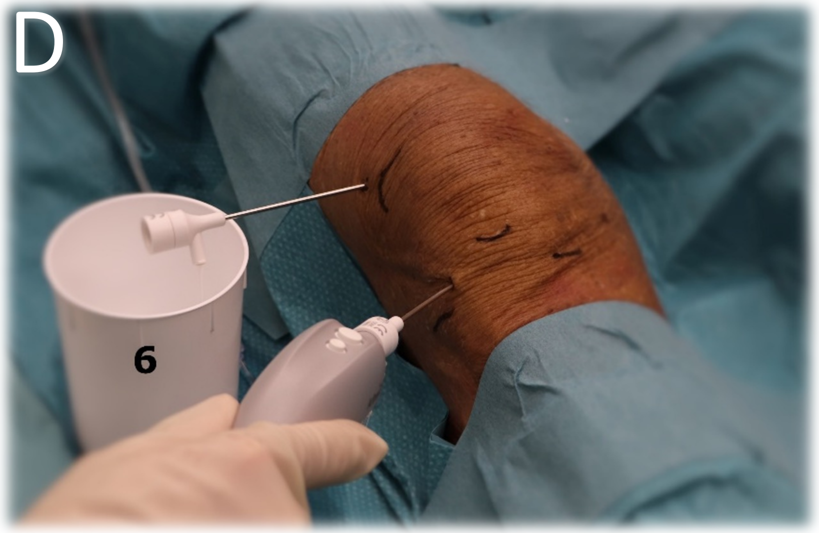

The Ankle

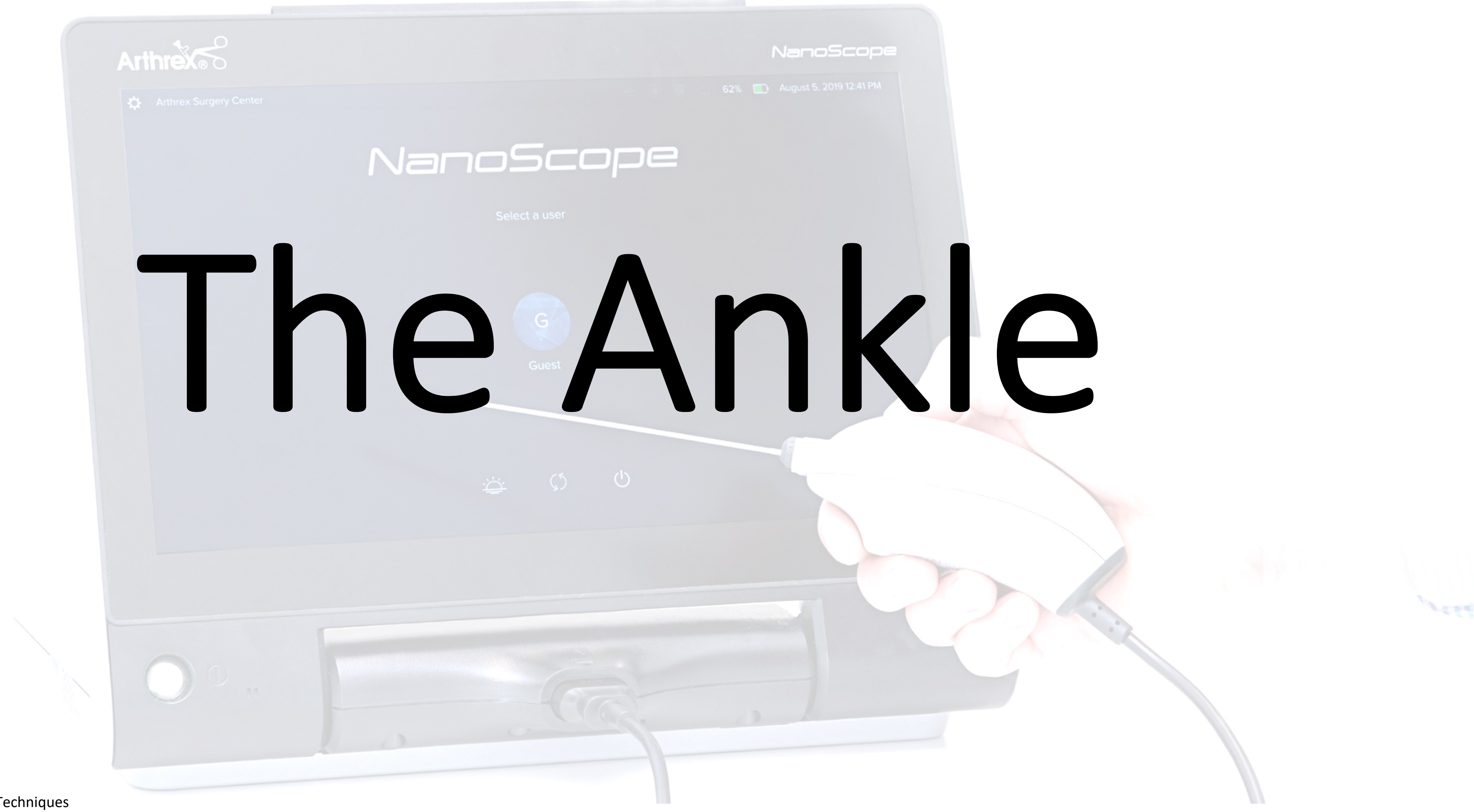

Walinga et al. 2022 Arthroscopy Techniques

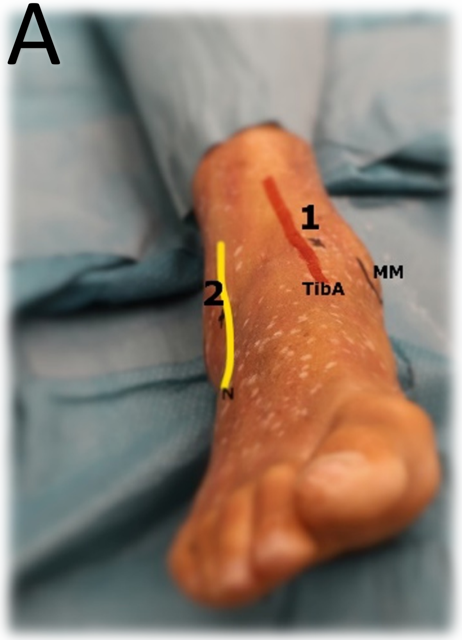

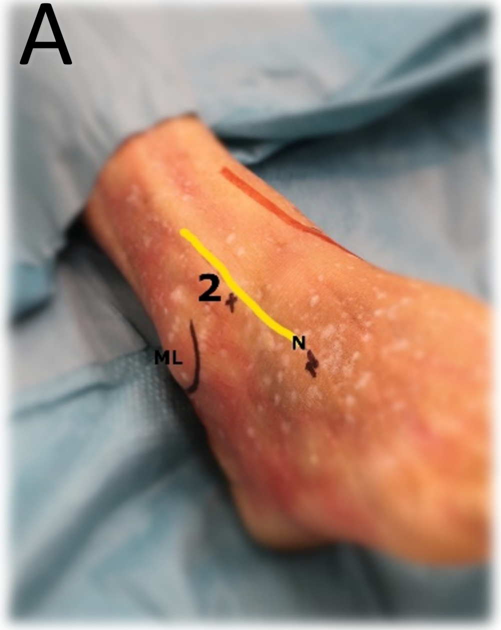

A. Position: the patient is placed supine. Inject local anesthesia (lidocaine 2%) to portal 1 and 2, and intra-articular. Make sure that the joint capsule is well anesthetized.
B. Portals:
    1) Anteromedial portal: medial to the tendon of the tibialis anterior (TibA) and lateral to the medial malleolus (MM). Make a portal between the tibialis anterior and the greater saphenous vein;
    2) Anterolateral portal: lateral to the peroneal tertius and superficial peroneal nerve (N) and medial to the lateral malleolus (ML).

C. Procedure portal 1: make a small 2.2-mm incision, and insert the blunt trocar with cannula into the joint (3). Connect an empty syringe to the cannula and aspirate synovial fluid for culture. Insert the needle arthroscopy through the cannula (4). Connect a 50cc syringe or a 500cc NaCl container coupled with a pressure bag to the cannula (5).

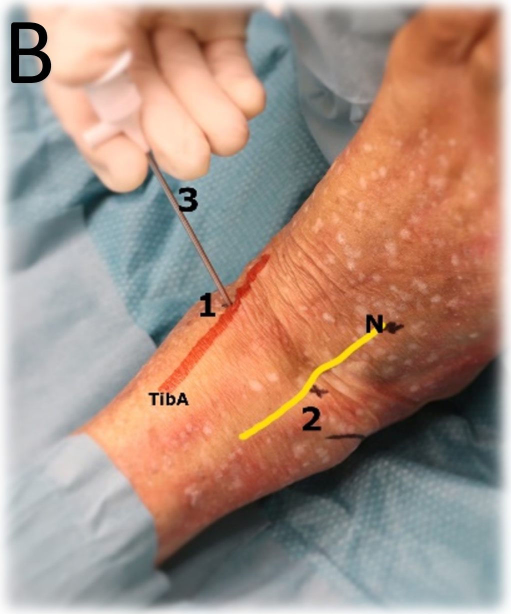

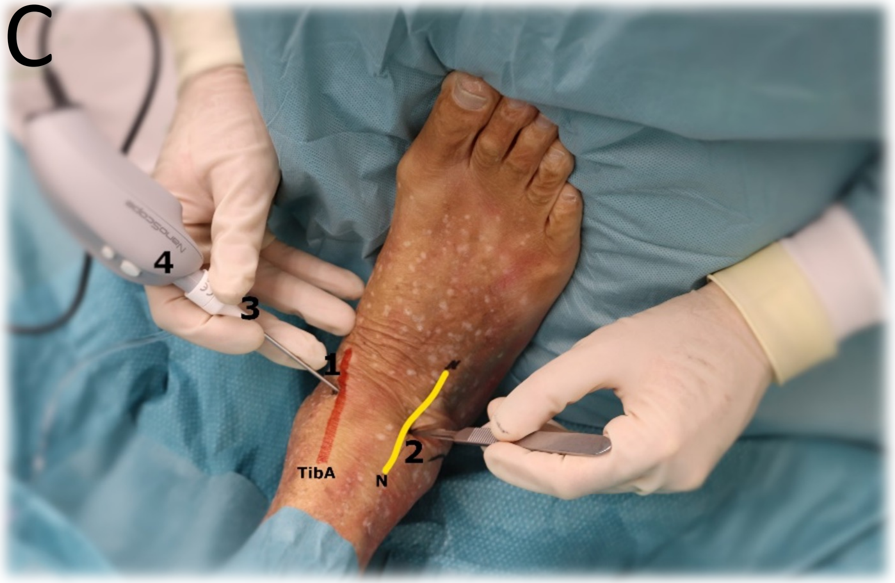

D. Procedure portal 2: make a small 2.2-mm incision under visualization with the needle arthroscope with a blunt trocar (6)

TIPS:
- When you remove the camera from the cannula, the flow of saline may be increased, so you can have a better lavage;
- It is possible to take biopsies for diagnostic purposes with a grasper;
- Slight non-invasive distraction may aid in portal placement.

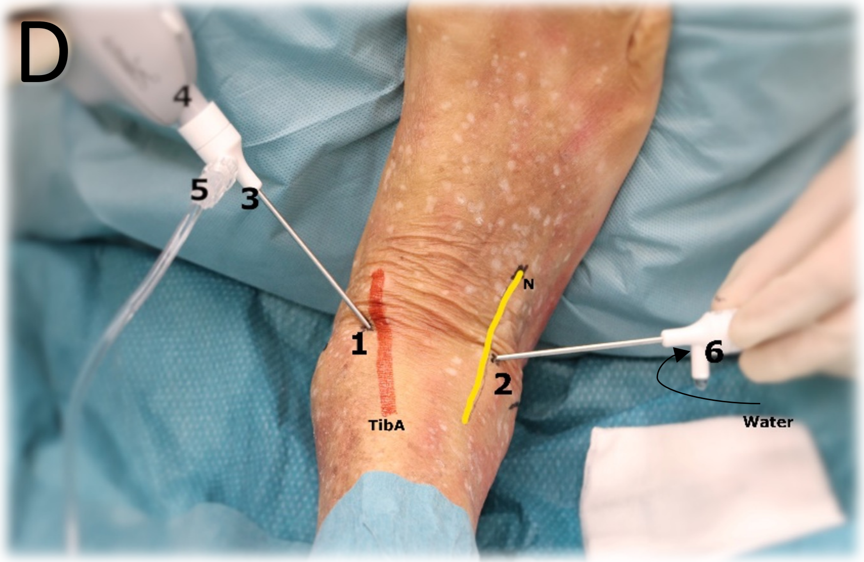

Walinga et al. 2022 Arthroscopy Techniques
